# Comprehensive Atomistic Simulations of Fischer–Tropsch in Outer Space: Astrocatalysis by Fe_13_–Supported Nanoclusters on SiO_2_

**DOI:** 10.1021/acs.jpcc.5c01472

**Published:** 2025-05-20

**Authors:** Gerard Pareras, Victoria Cabedo, Martin McCoustra, Albert Rimola

**Affiliations:** † Departament de Química, 16719 Universitat Autònoma de Barcelona, 08193 Bellaterra, Catalonia, Spain; ‡ Institute of Chemical Sciences, School of Engineering and Physical Sciences, 3120 Heriot-Watt University, Edinburgh EH14 4AS, Scotland

## Abstract

Catalytic processes are fundamental not only to terrestrial chemistry (e.g., in the synthesis of fuels, chemicals, and pharmaceuticals) but also to extraterrestrial chemistry, contributing to chemical reactions occurring in various astrophysical environments. In space, gas-phase reactions are limited due to sparse energy sources and the absence of a medium for energy dissipation, making heterogeneous catalysis on cosmic dust grains essential for driving chemical transformations. Iron nanoclusters (FeNCs) embedded on these grains present intriguing catalytic properties, especially for Fischer–Tropsch-type (FTT) reactions that synthesize interstellar organic compounds. This study investigates the formation of short-chain alcohols (CH_3_OH and CH_3_CH_2_OH) and hydrocarbons (CH_4_, CH_2_CH_2_ and CH_3_CH_3_) through a FTT mechanism using as astrocatalyst a realistic model of an Fe_13_ nanocluster supported on a silica (SiO_2_) surface (Fe_13_@SiO_2_) by characterizing the potential energy surfaces (PESs) and performing kinetics calculations. Comprehensive PESs grounded on density functional theory (DFT) reveal that direct CO dissociation on Fe_13_@SiO_2_ (required to form CH_3_CH_2_OH and CH_4_) is energetically unfavorable, but subsequent H_2_ addition facilitates CO bond cleavage, thus competing with the formation of CH_3_OH. Moreover, kinetic analysis indicates that C–O dissociation is more favorable than CH_3_OH synthesis, enabling chain-growing-based processes. Kinetics also predicts that the temperatures at which the FTT reactions can operate (i.e., above 100 K) are those available in protostellar regions and in evolved stages during a Solar-type planetary system formation (e.g., protoplanetary disks and primitive planetary environments).These findings offer a new proof on the feasibility of Astrocatalysis (namely, true chemical catalysis in astrophysical environments), in this case exerted by FeNCs, which partly alleviate stringent conditions required for FT on Earth, this way proposing a potential FTT-supported catalysis under milder conditions in astrochemical contexts.

## Graphical abstract

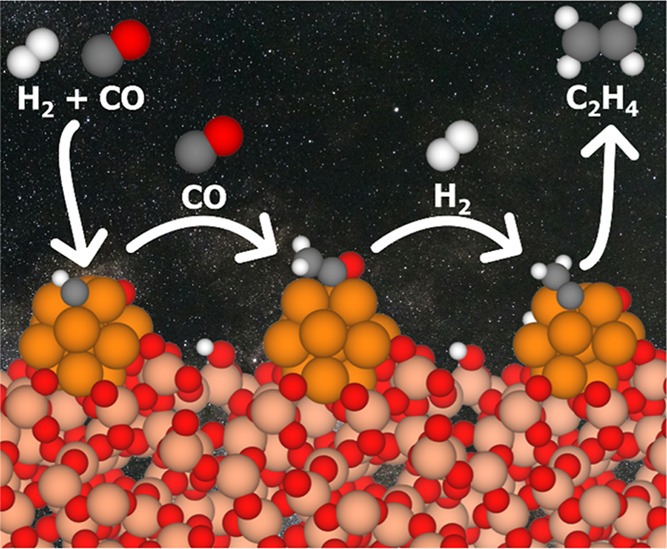

## Introduction

Catalytic processes are ubiquitous in our day-to-day life as they are essential not only for the synthesis of a wide range of products such as fuels, fertilizers, plastics and pharmaceuticals, but also for cleaning emissions from cars, power plants and industrial production. However, catalysis is not exclusively constrained to Earth; it also contributes to the chemistry of space, and more particularly heterogeneous catalysis. Cosmic chemical reactions can take place either in the gas phase or on the surfaces of solid-state dust grains. However, gas-phase reactions, although particularly relevant for radical-neutral and ion–molecule processes, present some limitations. One is that, due to scarce energy sources and the very low temperatures, reactions with activation energy are hampered. Another one is that, for barrierless and largely exergonic processes, the lack of a medium to dissipate the excess of the nascent reaction energies inhibits the occurrence of coupling reactions because the products dissociate back. Therefore, the presence of grain particles (of nanometre sizes) is of fundamental importance in the outer space chemical machinery (namely, presence and abundances of species and their synthetic routes) by facilitating chemical reactions occurring on their surfaces.

The composition of interstellar dust grains depends on the astrophysical environment where they are found, but the general picture is to present a core/mantle structure. The core is constituted by refractory materials while the mantle by ices of volatile species, both phases in an amorphous structural state. Ices are mostly dominated by H_2_O (water is the most abundant species in the solid phase), but they can also contain some amounts of CO, CO_2_, CH_3_OH, NH_3_, CH_4_. Relative to the refractory materials, silicates of the olivine and pyroxene families are among the most abundant ones.^1–5^ State-of-the-art investigations in Astrochemistry mostly focus on the chemistry occurring on the ices that cover the core of the dust grains, especially in the coldest regions of the interstellar medium (ISM, the region between stars, the matter of which forming the so-called interstellar clouds).^6–15^ Atomistic simulations based on quantum mechanical modeling is currently focused on the grain-surface chemistry of interstellar ices, revolutionizing the field, since the unprecedented information provided by the computations allows rationalizing for the first time the different mechanisms through which various interstellar molecules form in such regions, among them the so-called complex organic molecules (COMs), which represents the dawn of the organic chemistry.^16–19^ In this context, ices are advocated to have a catalytic effect on the reactions; however, by analyzing the roles of interstellar ices in such reactions, the term of catalysis is misused. Indeed, one role of the ices is as reactant concentrators. Densities in the ISM are very low (between 10 and 10^2^ and 10^4^–10^5^ atoms cm^–3^ in diffuse and dense interstellar clouds, respectively); therefore, adsorption of reactive species on the ice surfaces help concentrating them, favoring a subsequent encountering to react.^6–8,10,11^ Another role is as energy dissipators. As mentioned above, reactions in these cold regions lack a source of energy, and accordingly they must be barrierless or present low-energy barriers. Such processes are usually largely exergonic, and the ice surfaces serve to dissipate the surplus energy associated with the reaction, allowing the stabilization of the newly formed products.^9,12–15^ While it is true that these reactions are not feasible without the ice, if one sticks to the definition of a catalyst as a substance that increases the rate of a reaction without modifying the overall standard Gibbs energy change, ices, according to these roles, do not exhibit catalytic capabilities.

While the catalytic activity in ices is rather limited, refractory materials can present catalytic properties. Indeed, observations prove the presence of transition metals in the ISM, which can be incorporated into the dust grains.^20–22^ This is particularly the case of iron, the fifth most abundant element (and the first transition metal one) by mass in the solar abundance pattern, which is largely depleted in the gas phase and accordingly predominantly deposited in solid grains;^23–28^ for instance, in silicates, in which the [SiO_4_]^4–^ building blocks are combined with Mg^2+^ and Fe^2+^ to balance the charge. The mechanisms explaining Fe incorporation into the grains are largely debated, since it is difficult to determine when and how iron was sequestered from the gas phase to become part of the dust grain.^20^ Although being the most abundant refractory element together with magnesium and silicon, considering all the iron compounds (silicates, iron oxides/sulfides/phosphides, metallic inclusions) is insufficient to account for its estimated abundance in space. As a matter of fact, there is evidence pointing out to the existence of metallic iron aggregations (or inclusions) on the grains, which could be in the form of iron nanoclusters (Fe–NCs),^29–32^ on the basis that space weathering and sputtering of (Mg,Fe)-silicates give rise to silica (SiO_2_) rich material with nanoparticle Fe inclusions.^33,34^ Therefore, the presence of Fe–NCs opens up the possibility of structures that can be very versatile toward different catalytic processes.

Although the potentiality of Astrocatalysis (namely true chemical catalysis occurring in astrophysical environments, where solid-state systems presenting catalytic properties are present in the interstellar and circumstellar media or in extraterrestrial bodies), research focusing on the catalytic activity of dust grains is very scarce. Seminal experiments focused on the synthesis of hydrocarbons catalyzed by transition metal-containing dust analogues under simulated solar nebula conditions via Fischer–Tropsch-type (FTT) reactions^35–39^ demonstrating that true catalysis on cosmic grains can indeed occur. However, there is a lack of understanding of the mechanistic steps involved and the related energetics and kinetics that can explain such catalytic processes under the considered astrophysical conditions. Recent computational works have focused on describing reasonable reaction pathways for the formation of short-chain alcohols through FTT reactions using a simplified model of a single-atom iron (Fe-SA) on a silica (SiO_2_) surface (Fe-SA@SiO_2_).^40,41^ Mechanistic and kinetic data confirmed feasible FTT-synthesis of formaldehyde, methanol, methene, ketene, acetaldehyde, and ethanol, predicting the need of energy sources achievable in astrophysical regions with temperatures higher than 200 K. In the same way, CH_3_OH synthesis via FTT processes on cosmically occurring iron sulfide surfaces has been theoretically investigated, showing less efficient synthetic routes, in which temperatures of at least 500 K are needed.^39^ These theoretical data allowed rationalizing for the first time how and where FTT-catalyzed processes can take place in space. However, as mentioned above, Fe can greatly be found forming metal aggregations in the grains rather than as single atoms. Therefore, the scope of this work is to study the formation of short-chain alcohols and hydrocarbons by adopting a FTT scheme catalyzed by metal Fe-NC on SiO_2_ surfaces (hereafter referred to as Fe-NC@SiO_2_), providing an accurate mechanistic study that unveils the energetics required for the formation of different FTT products, i.e., CH_3_OH, CH_3_CH_2_OH, CH_4_, CH_2_CH_2_ and CH_3_CH_3_. Moreover, kinetic calculations serve to further comprehend the catalytic activity of the Fe-NC@SiO_2_ system and predict in which astrophysical environments the reactions can be operative. The framework of the investigated processes aligns well with recent studies dedicated to the size effect of iron nanoparticles (Fe-NPs) in FT synthesis.^42^ De Jong et al.^43,44^ demonstrated that reducing the size of Fe-NPs from 7 to 2 nm significantly enhances the initial catalytic activity in FT reactions, in consistency with the size-dependency of the catalytic activity of nanomaterials, which in turn are distinct from their bulk counterparts.^45–47^ Additionally, SiO_2_ surfaces are widely recognized as inert solid supports, and hence frequently used to improve the physical properties of SiO_2_-supported Fe-catalysts through structural promotion.^48,49^ Thus, our findings are not only pertinent to the field of astrochemistry but can also offer valuable insights into FT reaction mechanisms, potentially advancing the synthesis of alcohols and hydrocarbons on Earth.

## Methodology

### Computational Details

All the calculations were performed adopting a periodic approach and using the CP2K package.^50^ Characterization of the potential energy surface (PESs) requires determining the structures and the energetics of the stationary points. For geometry optimizations, the semilocal PBEsol functional was used,^51^ along with the Grimme’s D3­(BJ) correction to include dispersion forces.^52^ A double-ζ basis set (DZVP-MOLOPT-SR-GTH Gaussian basis set) was adopted for all the atom types, combined with a cutoff set at 500 Ry for the plane wave auxiliary basis set.^50,53^ The Goedecker–Teter–Hutter pseudopotentials^54^ were used to describe core electrons, while a mixed Gaussian and plane-wave (GPW) approach^55^ was employed for valence electrons. The energies of the stationary points were refined by performing single point calculations onto the PBEsol-optimized geometries at the hybrid B3LYP functional theory level,^56,57^ with the D3­(BJ) dispersion correction and using the triple-ζ (TZVP) basis set. Note that the auxiliary density matrix method (ADMM)^58,59^ was used for the exact exchange when performing calculations with hybrid functionals. The selection of B3LYP-D3­(BJ) method for the energetic refinement was based on a consistent benchmarking study. This was carried out by using the ORCA package,^60^ and it consisted in finding the DFT method that better describes the electronic structure of a Fe nanocluster of 7 atoms (Fe_7_), taking as a paradigmatic parameter the energy difference between the triplet and the singlet electronic states, corresponding to the ground- and first-excited states, respectively. Results calculated at the DLPNO–CCSD­(T)/cc-pVDZ level of theory^61–63^ were taken as the reference values. The tested functionals were PBE,^64^ B3LYP,^56,57^ PBE0,^65,66^ BHLYP^56^ and wB97X,^67^ in all cases with the TZVP basis set and D3­(BJ) dispersion corrections. Results (reported in the Table S1) indicate that the two functionals showing the best performance are B3LYP and PBE0, with a relative error per atom of 8.6% and 8.1% respectively.

B3LYP was ultimately chosen as the functional, as it had already been employed in our previous studies on astrocatalytic FTT reactions,^40,41^ demonstrating very good agreement with DLPNO–CCSD­(T)/cc-pVDZ results. Its use also facilitates consistent comparisons across related studies. Moreover, the B3LYP-D3­(BJ)//PBEsol-D3­(BJ) computational scheme proved to be a cost-effective approach for the systems and reactions investigated.

The climbing image nudged elastic band (CI-NEB)^68^ technique implemented in CP2K^50^ was used to search for transition states, which were also calculated at the B3LYP-D3­(BJ)//PBEsol-D3­(BJ) theory level. Energy barriers were calculated as

1 $${\Delta E}^{‡}=E_{TS}-E_{GS}$$

2 $${\Delta U}^{‡}={\Delta E}^{‡}+\Delta ZPE$$

3 $${\Delta G}_{T}^{‡}={\Delta E}^{‡}+{\Delta G}_{T}$$

where Δ*E* ^‡^ stands for the potential energy barrier, and *E*_TS_ and *E*_GS_ for the absolute potential energies of the transition states and the previous local minima, respectively. Δ*U* ^‡^ represents the zero-point energy- (ZPE) corrected barrier (in which ΔZPE refers to the contribution of the ZPE corrections to Δ*E* ^‡^), and Δ*G*_T_^‡^ for the Gibbs energy barrier at a given temperature, in which Δ*G*_T_ refers to the contribution of the Gibbs corrections to Δ*E* ^‡^ calculated as

4 $${\Delta G}_{T}=\Delta H-T\Delta S$$

where Δ*H* stands for enthalpy, *T* the temperature and Δ*S* the entropy,

The nature of the stationary points of the reactions was validated by calculating the harmonic frequencies (minima for reactants, intermediates and products, and first-order saddle points showing only one imaginary frequency for transitions states). Vibrational harmonic frequencies were calculated at the PBEsol-D3BJ/DZVP-optimized structures using the finite differences method as implemented in the CP2K code.^50^ To minimize the computational cost, a partial Hessian approach was employed. Consequently, vibrational frequencies were computed solely for a subset of the entire system, comprising the surface atoms participating in the reaction and the reactive species.

The catalytic performance of the simulated FTT processes was investigated through reaction rate kinetic calculations. To this aim, a rate constant associated with each elementary barrier was calculated using the Rice–Ramsperger–Kassel–Marcus (RRKM) theory,^69^ a microcanonical transition state theory that assumes that the phase space is statistically populated. In this RRKM treatment, tunnelling effects were taken into account by adopting the unsymmetrical Eckart potential barrier model.^70^ For the calculation of the rate constants, we used the calculated vibrational frequencies as degrees of freedom in the sum of states. Although we calculated a partial Hessian matrix to derive a set of vibrational modes, they are those directly involved in the reaction, and accordingly those that have a direct impact on the rate constants. The rest of the vibrational modes, which are not accounted for, belong to the surface inner layers and are assumed to have a negligible influence in the chemical reactions and by extension the rate constants.^71^ These kinetic calculations were performed with a freely available in-house program, in which the RRKM algorithms were implemented for grain–surface processes.^72^

### Surface Catalyst Model

The surface catalyst model consists of a periodic SiO_2_ slab surface model obtained from Ugliengo et al.,^73^ where a 13-atom Fe nanocluster (Fe_13_) has been incorporated on its surface (hereafter referred to as Fe_13_@SiO_2_), and relaxed the final structure reaching the ground state. The selected amorphous SiO_2_ slab presents a low silanol surface density (1.5 SiOH nm^–2^) and contains 187 atoms per unit cell. Upon cell optimization at the PBEsol level, the cell parameters are *a* = 13.142 Å, *b* = 13.056 Å and *c* = 49.357 Å and α = β = 90° degrees and γ = 90.25° with a thickness of 17.491 Å and an empty space between slab replicas of 31.870 Å in the *z* direction. The corresponding Brillouin zone was sampled at the Γ point (Figure 1A,B).

**Figure 1:**
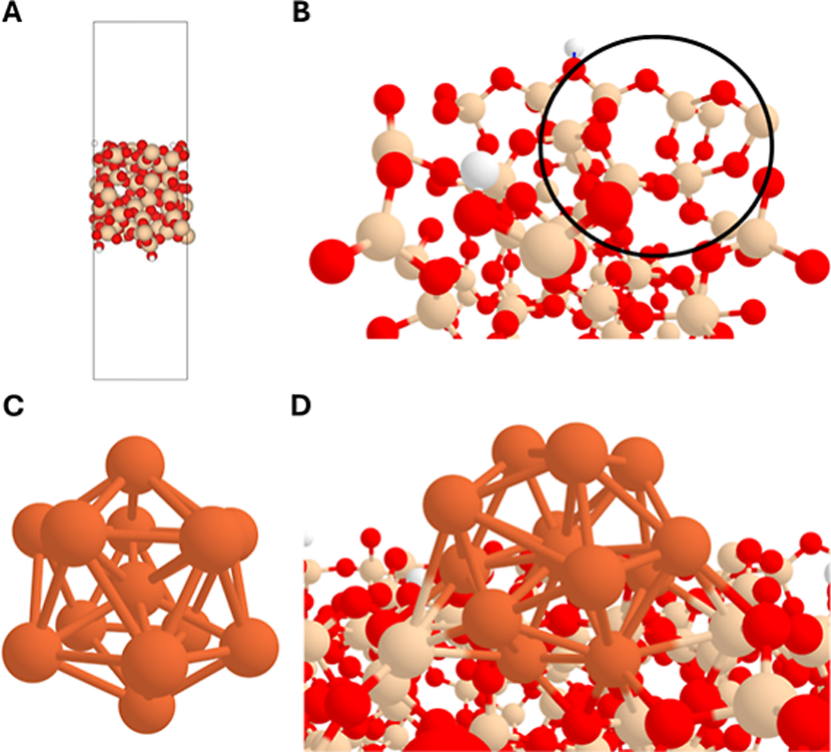
(A) PBEsol-D3­(BJ)-optimized structure of the periodic SiO_2_ surface. The unit cell is highlighted by the black box. (B) Zoom in on the SiO_2_ surface. The black circle highlights the region where the Fe_13_ is attached to. (C) PBE-optimized structure of the Fe_13_ cluster. (D) PBEsol-D3­(BJ)-optimized structure of the Fe_13_@SiO_2_ system. Color-coding: white, H atoms; red, O atoms; beige, Si atoms; and orange, Fe atoms.

The selection of the Fe_13_ nanocluster was based on its individual stability. Fe_13_ is considered a “magic cluster” as it shows to be the most stable cluster from Fe_02_ to Fe_15_ systems. Other clusters showing similar stabilities are Fe_06_, Fe_07_ and Fe_08_.^74,75^ As mentioned above, Fe_07_ was the cluster system to conduct the preliminary benchmarking study. The Fe_13_ cluster was optimized at the PBE level previous attachment on the SiO_2_ surface, as it has been reported its icosahedral motif does not undergo geometrical changes upon PBE optimization (Figure 1C). For the sake of consistency, different Fe_13_ electronic states were computed, showing that its electronic ground state is high spin state, which aligns well with the benchmarking results at CCSDT. Considering that all the Fe atoms have a formal oxidation state of 0, each of them with two unpaired electrons, the final Fe_13_ electronic multiplicity is 27.

The Fe_13_@SiO_2_ catalyst was build up by directly attaching Fe_13_ on the SiO_2_ surface and relaxing the final geometry. The optimized structure (Figure 1D) shows a slightly distorted Fe_13_ cluster. Fe–Fe distances in isolated Fe_13_ are between 2.38 Å–2.56 Å with and average value per bond distance of 2.41 Å, while in the Fe_13_@SiO_2_ system Fe–Fe distances are between 2.29–2.86 Å, but with an average bond distance of 2.46 Å. Thus, although the Fe–Fe bond distances are affected by the interaction with the SiO_2_ surface, the final average bond distances are very similar, matter of fact that Fe_13_ structure reorganizes keeping the icosahedral motif. The bonds showing an increase of their length are the ones in direct contact with the surface, in agreement with the work of Gueddida et al.^75^ where a variation of the average bond distances of 0.02 Å was reported. The principal interactions between Fe_13_ and the SiO_2_ surface are through Fe–O and Fe–Si bonds belonging to siloxane (Si–O–Si) groups. No interactions with silanol groups are observed due to the low silanol density of the SiO_2_ surface model.

## Results

### Proposed Catalytic Cycle

The mechanisms of Fischer–Tropsch processes have been broadly studied over the past 20 years, in which dedicated DFT calculations have become increasingly popular to evaluate mechanistic proposals.^39,40,76–84^ However, still today, there is a strong debate on which mechanism predominates. One can distinguish between three main reaction mechanisms, the carbide mechanism, the CO-insertion mechanism and the hydroxycarbene mechanism. However, in all the mechanisms, the CO dissociation is considered a key step as it forms CH_x_* species, which act as monomers and/or chain initiators. In this work, we consider the reaction to advance with the lowest number of H_2_ and CO molecules because, first, although H_2_ and CO are two of the most abundant molecules in space, their relative abundancies are very small compared to terrestrial standards, thus providing a realistic mechanistic study to comprehensively understand and predict if the studied processes are operative in diverse astrophysical environments. The proposed catalytic cycle is shown in Figure 2. The studied mechanism involves three different processes: (i) the first insertion of a CO molecule (CO—First insertion), which can lead to the formation of methanol, (ii) the second insertion of a CO molecule after dissociation of the first CO forming the CH_2_ chain initiator forms (CO—Second insertion), which can lead to the formation of ethanol, and (iii), the methanation mechanism, in which the CH_2_ can be transformed into CH_4_.

**Figure 2:**
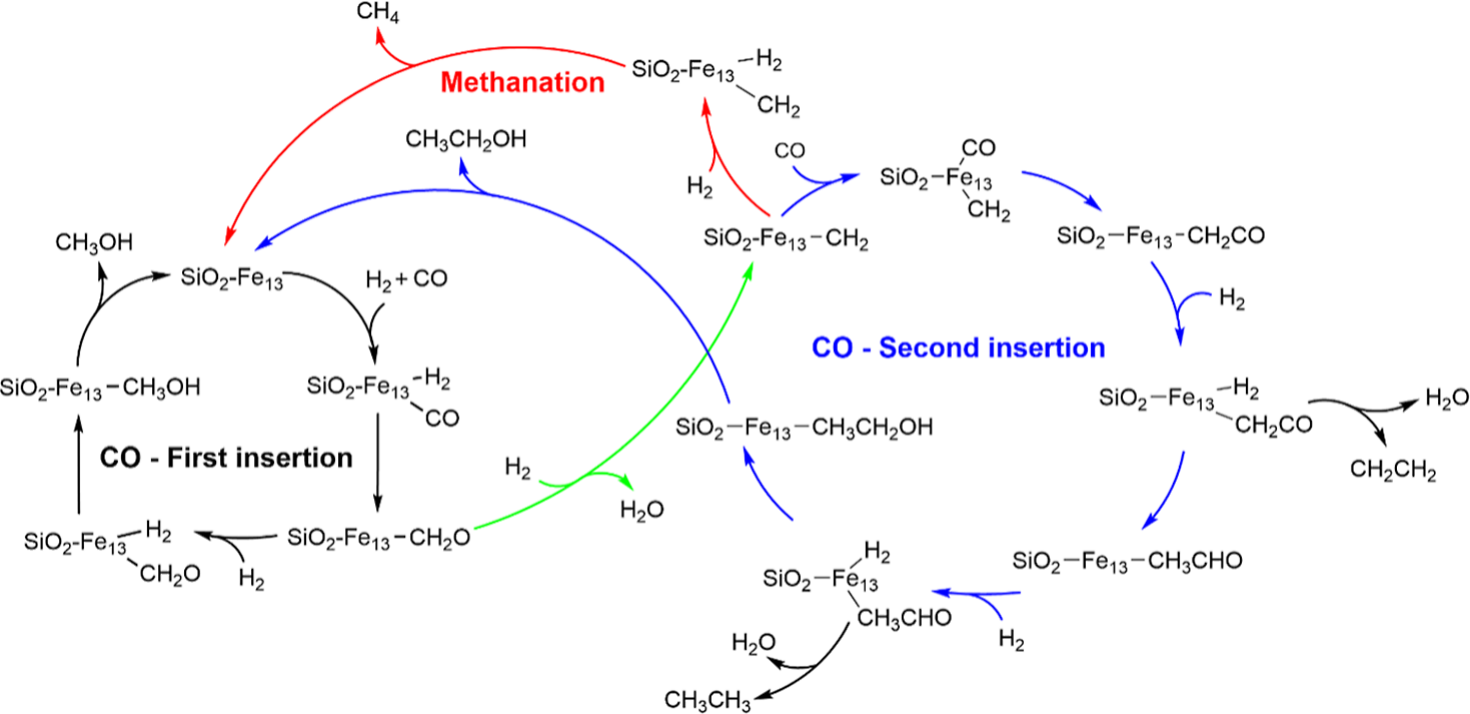
Proposed catalytic cycle showing the FTT processes considered in this work.

### CO—First Insertion Mechanism

Figure 3 shows the calculated structures and the PESs involved in the first CO insertion. The mechanism begins with the adsorption of H_2_ molecule on Fe_13_@SiO_2_, which undergoes spontaneous homolytic cleavage on the NC surface, leading to the exoergic formation (−13 kcal mol^–1^ with respect to the asymptote) of the [H_2_] intermediate (see Figure 3A,F). Subsequently, CO adsorbs at the top site of Fe_13_, forming the [H_2_+CO] intermediate, with a relative energy of −18.9 kcal mol^–1^ with respect to the asymptote. At this stage, two possible pathways emerge: direct CO dissociation to form [C + O], or initial hydrogenation to form the [H + HCO] intermediate. The direct dissociation of CO (TS­[C + O] of Figure 3B,F) involves a high energy barrier of 90.2 kcal mol^–1^, which is consistent with previous studies highlighting the high energy cost for CO dissociation, although it is highly exergonic, with the [C + O] dissociation product being at −93.4 kcal mol^–1^. In contrast, the hydrogenation of CO is found to be energetically more favorable, with an energy barrier of 9.3 kcal mol^–1^, leading to the exergonic formation of the [H + HCO] intermediate at −28.2 kcal mol^–1^. The observed partial activation of the CO molecule is insufficient to promote direct C–O bond dissociation due to electronic effects, as evidenced by the substantially higher activation energy compared to the hydrogenation barrier.

**Figure 3:**
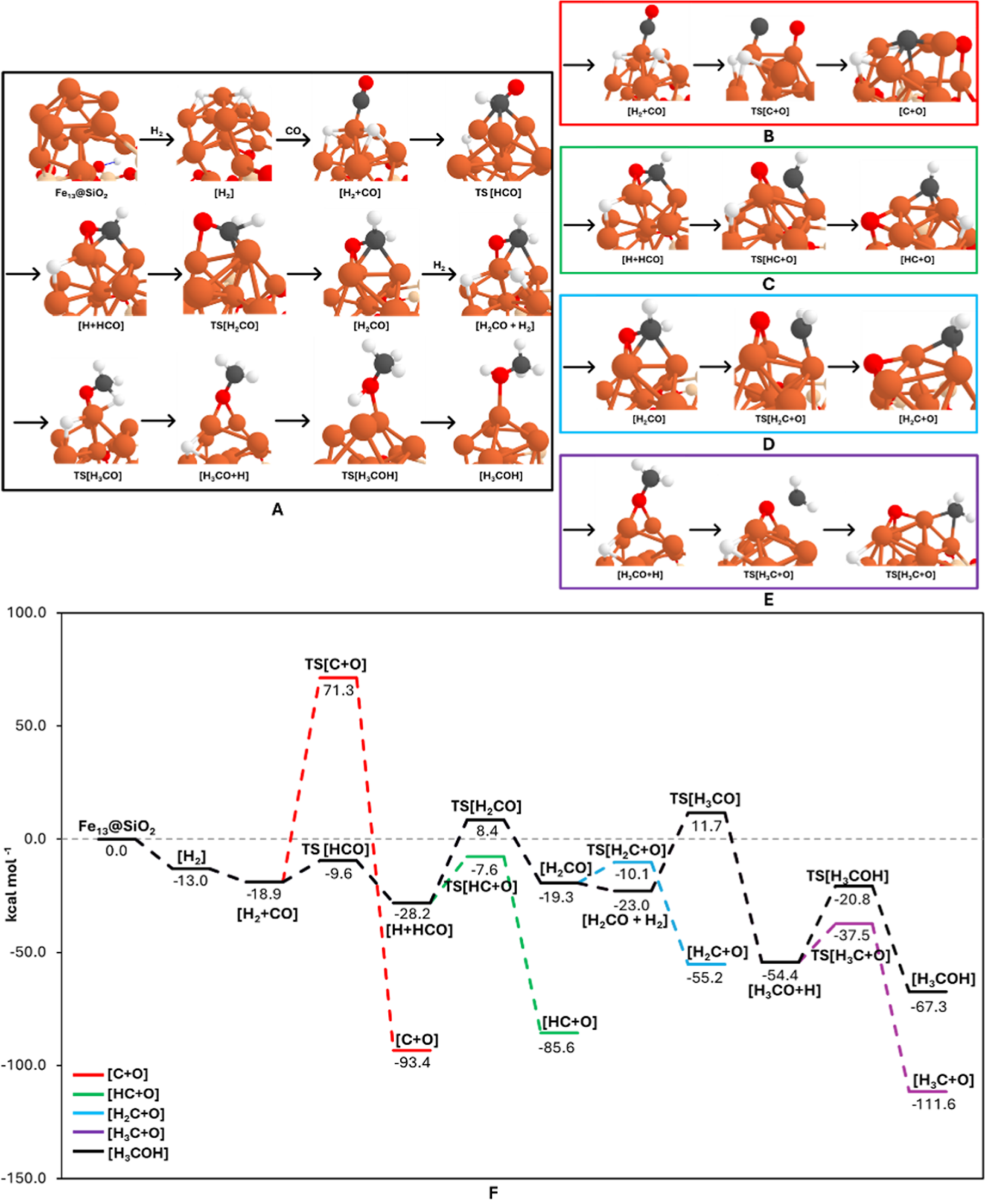
(A–E) PBEsol-D3­(BJ)-optimized geometries of the structures involved in the first CO insertion mechanism, depicting the H_2_ additions to CO that lead to methanol formation (A) and the relevant CO dissociations (B–E), (F) B3LYP-D3­(BJ)//PBEsol-D3­(BJ)-potential energy surfaces (in kcal mol^–1^) for the processes involved in the first CO insertion mechanism (pathways A–E). Color-coding: white, H atoms; gray, C atoms; red, O atoms; beige, Si atoms; and orange, Fe atoms.

From [H + HCO], two competitive pathways are possible: either the dissociation of the HCO species forming [HC + O], or further hydrogenation to form [H_2_CO]. The dissociation barrier is of 20.6 kcal mol^–1^, the resulting product being stabilized by −85.6 kcal mol^–1^. In contrast, the hydrogenation has a higher energy barrier (36.6 kcal mol^–1^) and is endergonic with respect to [H + HCO], with the final [H_2_CO] product at −19.3 kcal mol^–1^ with respect to the initial asymptote. Such a reduction in the CO dissociation barrier through hydrogenation was expected, as already noted in previous studies as a plausible way to achieve it.^83,84^ The 16 kcal mol^–1^ difference between the dissociation and hydrogenation barriers suggests that, at low temperatures, the dissociation pathway is favored over hydrogenation. Moreover, [H_2_CO] can also dissociate into [H_2_C + O] through an intrinsic barrier of 9.2 kcal mol^–1^, with the dissociated product at −55.2 kcal mol^–1^. Note that the dissociated product includes a free oxygen coordinated on the metal cluster, this can further react with hydrogen to form H_2_O avoiding the poisoning of the Fe centers in the cluster. This process has been also calculated, and the relevant energies are reported in the Supporting Information, in Tables S12 and S13.

Despite that the C–O dissociation barriers are energetically more favorable, to go on with the CH_3_OH formation through the first CO-insertion path, the adsorption of a second H_2_ molecule takes place, which (like the first H_2_) undergoes a spontaneous homolytic cleavage with an exoergic intermediate at −23 kcal mol^–1^ ([H_2_CO + H_2_] intermediate of Figure 3A). Then, [H_2_CO + H_2_] can lead to [H_3_CO + H] through an H addition to H_2_CO, which requires overcoming a barrier of 34.7 kcal mol^–1^, with the product at −54.4 kcal mol^–1^. Note that the hydrogenation of the oxygen instead of the carbon moiety was ruled out as it has been described to be energetically unfavorable.^39–41,80,81^ Finally, subsequent hydrogenation leads to the formation of the final [H_3_COH], with a barrier of 33.6 kcal mol^–1^ and an exergonic final product at −67.3 kcal mol^–1^. [H_3_CO + H] also presents the possibility of the C–O dissociation from the H_3_CO species, with an energy barrier of 16.9 kcal mol^–1^ and the [H_3_C + O] dissociated product being largely exergonic. Interestingly, this dissociation barrier is still lower than the relevant hydrogenation but higher than the C–O dissociation barrier in H_2_CO.

Our calculations indicate that the direct reaction of CO with H_2_ preferentially leads to the formation of the chain initiator CH_2_, with methanol formation being energetically less favorable. Here, the final formation of methanol is energetically less favorable because the activation energies for breaking the CO bond, even at the HCO step, are lower than the activation energies for the subsequent hydrogenation steps. Consequently, the molecule will break to form the chain initiator CH_2_ before reaching the final formation of methanol. This suggests that the FTT reaction catalyzed by Fe_13_@SiO_2_ is more likely to proceed through a polymerization scheme before yielding CH_3_OH, which aligns well with industrial FT processes, where alkenes are the primary products, and alcohols are byproducts.^79^ Accordingly, the next section focuses on the newly formed CH_2_ chain initiator and examine the second CO insertion.

### CO—Second Insertion Mechanism

Figure 4 shows the calculated structures and the PESs involved in the CO—second insertion, which begins with the newly formed CH_2_ acting as a chain initiator (the [CH_2_] reactant structure in Figure 4A, which serves as the zeroth energy reference asymptote). The CH_2_ moiety was selected as this species can be formed either through the direct dissociation of CH_2_O or via hydrogenation of the dissociated HC intermediate. Notably, the latter reaction has also been computationally evaluated, and the corresponding energetics are provided in Tables S14 and S15 of the Supporting Information.

**Figure 4:**
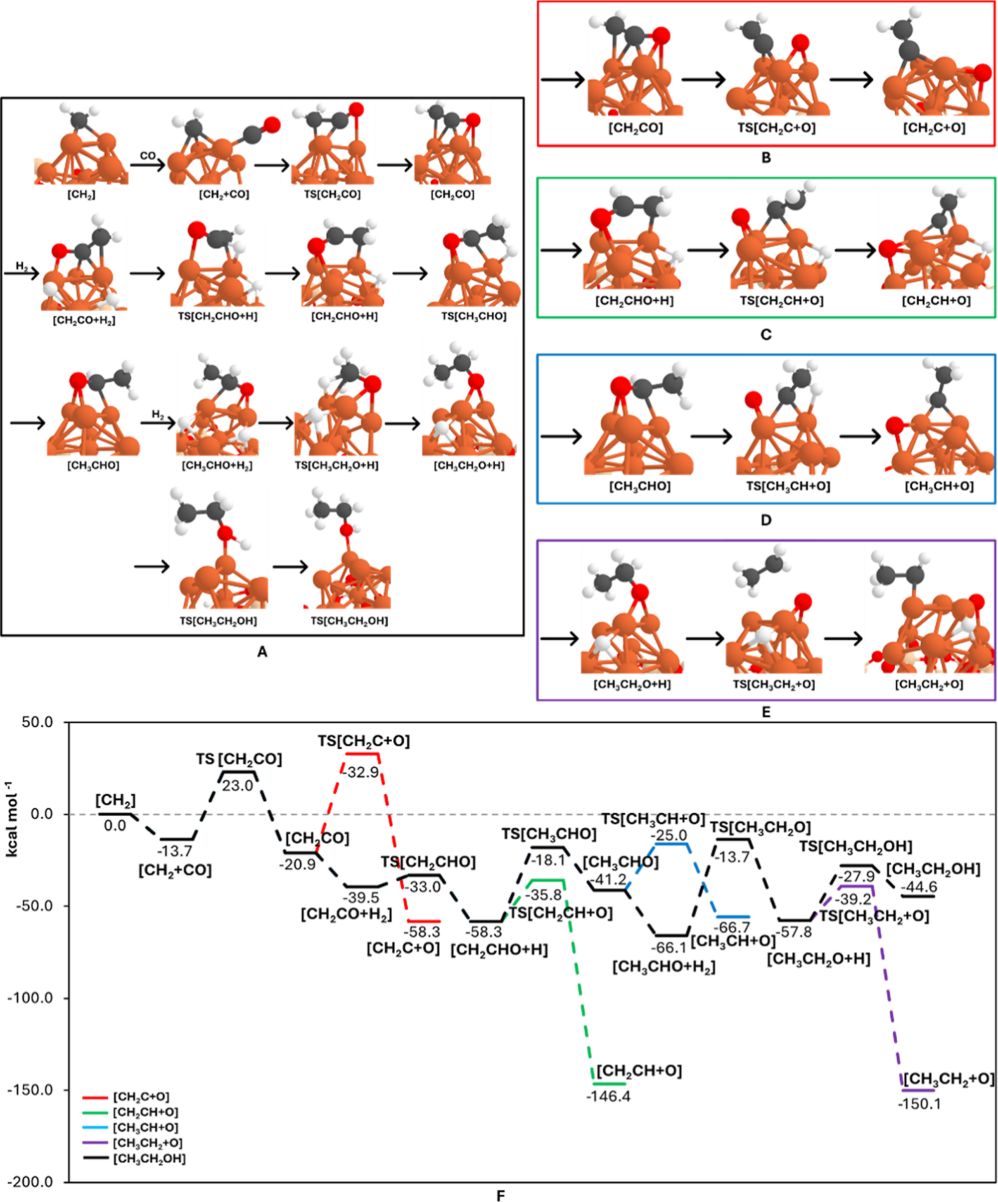
(A–E) PBEsol-D3­(BJ)-optimized geometries of the structures involved in the second CO insertion mechanism, depicting the H2 additions leading to ethanol formation (A) and the relevant CO dissociations (B–E). (F) B3LYP-D3­(BJ)//PBEsol-D3­(BJ)-potential energy surfaces (in kcal mol^–1^) for the processes involved in the second CO insertion mechanism (pathways A–E). Color-coding: white, H atoms; gray, C atoms; red, O atoms; beige, Si atoms; and orange, Fe atoms.

Following the chain growing scheme, a second CO molecule is adsorbed on [CH_2_], leading to the intermediate [CH_2_ + CO] with a relative energy of −13.7 kcal mol^–1^. Direct CO dissociation is not considered due to its high energy requirements, as previously reported. The next step involves the direct coupling of CH_2_ with CO, which requires overcoming a barrier of 36.7 kcal mol^–1^ and that exergonically forms the ketene [CH_2_CO] intermediate. To explore all potential reaction pathways, the different C–O dissociations from the reaction intermediates have been considered. In [CH_2_CO], the C–O cleavage has an intrinsic energy barrier of 53.7 kcal mol^–1^, resulting in the highly exergonic product [CH_2_C + O] (see Figure 4B,F). This direct C–O breaking is highly energy-demanding, suggesting that CO hydrogenation is required to facilitate it. Accordingly, a new H_2_ molecule is favorably adsorbed, which spontaneously dissociates into the two H atoms, forming the intermediate [CH_2_CO + H_2_] (see Figure 4A,F).

The first hydrogenation of ketene, with a barrier of 6.5 kcal mol^–1^, leads to the exergonic intermediate [CH_2_CHO + H]. It is worth noting that hydrogenation at the terminal carbon has been previously reported to require higher energy.^39–41,84^ At this juncture, the reaction can proceed via two different routes: either C–O dissociation of CH_2_CHO from [CH_2_CHO + H] or further hydrogenation of CH_2_CHO to form acetaldehyde (CH_3_CHO). The former process leads to the exergonic formation of [CH_2_CH + O] with a barrier of 22.5 kcal mol^–1^ (see Figure 4C,F), while the latter has a higher barrier of 40.2 kcal mol^–1^, resulting in the endergonic formation of the [CH_3_CHO] product (see Figure 4A,F).

Despite that formation of [CH_3_CHO] is relatively disfavored, its fate, which can lead to the final formation of ethanol, has also been considered to have a comprehensive description of the FTT processes. The newly formed acetaldehyde [CH_3_CHO] intermediate can either undergo a C–O bond dissociation forming the exergonic [CH_3_CH + O] product with a barrier of 25.3 kcal mol^–1^ (see Figure 4D,F), or can involve a new H_2_ adsorption, forming the [CH_3_CHO + H_2_] intermediate at −66.1 kcal mol^–1^, in which H_2_ again spontaneously splits into two H atoms. The H additions to acetaldehyde results in the endergonic formation of the [CH_3_CH_2_O] intermediate (first addition) with a barrier of 52.3 kcal mol^–1^, and the final formation of [CH_3_CH_2_OH] (second addition), forming ethanol as an endergonic product through a barrier of 29.9 kcal mol^–1^ (see Figure 4A,F).Finally, the [CH_3_CH_2_O] intermediate can also undergo a C–O bond cleavage forming the exergonic [CH_3_CH_2_ + O] product through an energy barrier of 18.6 kcal mol^–1^ (see Figure 4E,F).

### Alkane and Alkene Formation

We investigated three distinct scenarios for the formation of short-chain alkanes (CH_4_ and C_2_H_6_) and alkenes (C_2_H_4_). These processes occur either through the introduction of an H_2_ molecule or when hydrogen is already present in the system.

The formation of methane (CH_4_) involves the reaction of the chain initiator CH_2_ with an H_2_ molecule instead of CO (see methanation mechanism of Figure 2). The PES and the associated reaction mechanism are presented in Figure 5A,B. This reaction, known as methanation in the context of FT synthesis, is typically undesirable because it terminates the FT polymerization process. However, understanding methanation is critical in astrophysical contexts due to the abundance of methane in certain environments, such as the liquid methane and ethane lakes on Titan, Saturn’s largest moon.

**Figure 5:**
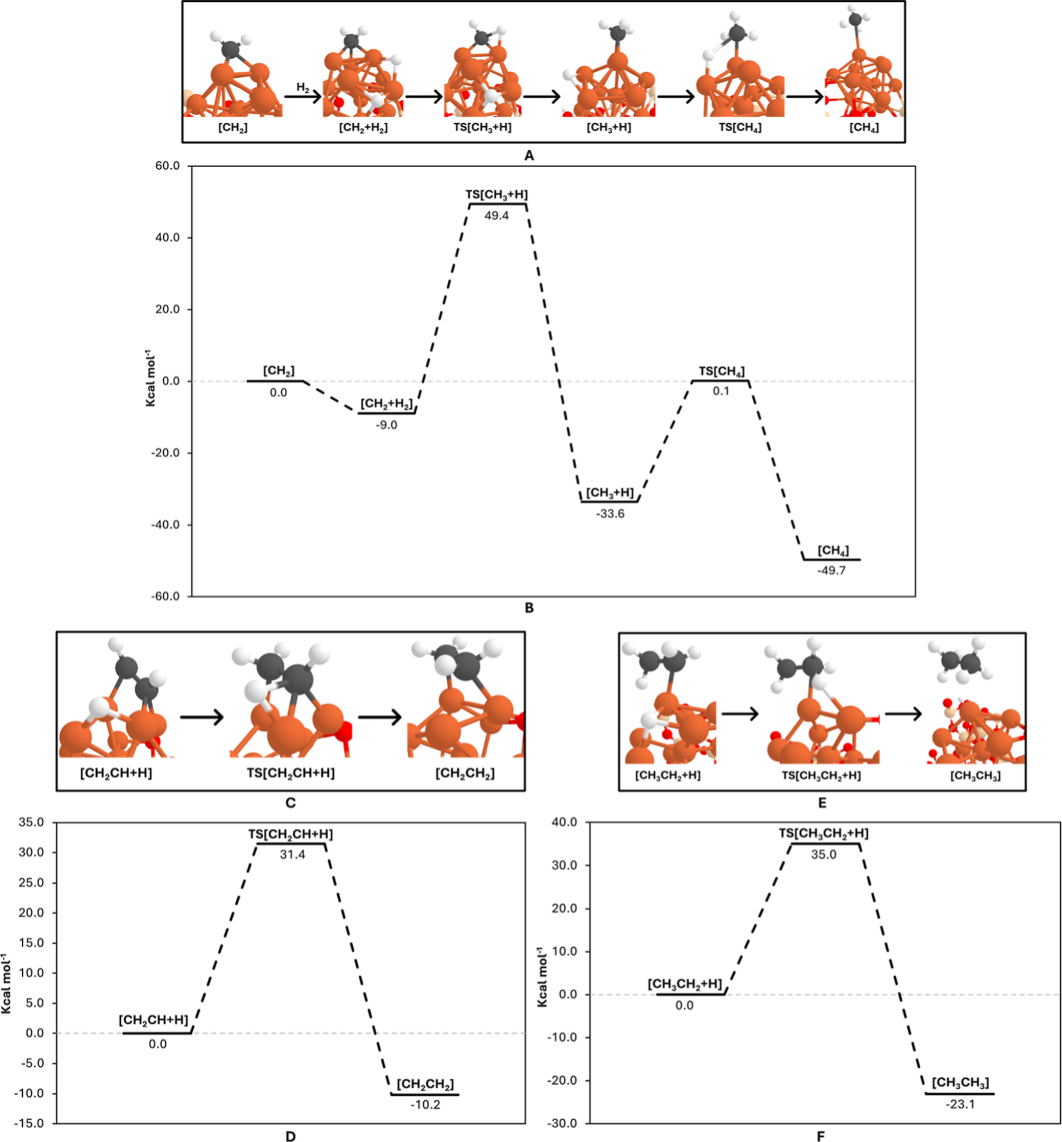
(A) PBEsol-D3­(BJ)-optimized geometries of the structures involved in the methanation (formation of CH_4_) mechanism. (C) Formation of CH_2_CH_2_. (E) Formation of CH_3_CH_3_. (B,D,F) Associated potential energy surfaces (in kcal mol^–1^) calculated at the B3LYP-D3­(BJ)//PBEsol-D3­(BJ) (B) theory level. Color-coding: white, H atoms; gray, C atoms; red, O atoms; beige, Si atoms; and orange, Fe atoms.

The adsorption of H_2_ on the nanocluster occurs spontaneously, leading to its dissociation into two H atoms. This creates an exergonic [CH_2_ + H_2_] intermediate. The first hydrogen transfer to CH_2_ results in the exergonic formation of CH_3_, shown as the [CH_3_ + H] intermediate in Figure 5. This step proceeds via an activation energy barrier of 58.4 kcal mol^–1^. A second hydrogen addition then forms CH_4_ through a lower activation energy barrier of 33.8 kcal mol^–1^.

Although both steps are exergonic, the CH_2_ moiety is particularly stabilized on the nanocluster, resulting in a significant energy barrier for the first hydrogen addition. This stabilization makes the energy barrier for the second CO insertion mechanism, although relatively high (36.7 kcal mol^–1^), lower than that for the direct reaction between CH_2_ and H. This indicates that CO insertion, which drives chain growth, is energetically more favorable than methane synthesis.

In the previously described second CO-insertion mechanism, we considered various pathways where CO dissociates. Here, we focus on the possible hydrogenation of the resulting intermediates following C–O bond cleavage, leading to the formation of C_2_H_4_ (ethylene) and C_2_H_6_ (ethane). Specifically, we analyzed the reaction steps involving [CH_2_CH + O] and [CH_3_CH_2_ + O] (intermediate favorably formed during the second CO insertion path), where an available H atom can hydrogenate these dissociated intermediates to produce ethylene and ethane.

The mechanisms and calculated PESs for ethylene and ethane formation are depicted in Figure 5C–F. In both cases, the hydrogenation processes involve relatively high activation energies (Δ*U* ^‡^[CH_2_CH_2_] = 31.4 kcal mol^–1^ and Δ*U* ^‡^[CH_3_CH_3_] = 35.0 kcal mol^–1^). However, both reactions are exergonic, with reaction energies of Δ*U*_rx_[CH_2_CH_2_] = −10.2 kcal mol^–1^ and Δ*U*_rx_[CH_3_CH_3_] = −23.1 kcal mol^–1^.

Interestingly, the hydrogenation steps are strongly influenced by the stabilization of both the intermediates and hydrogen atoms on the nanocluster. Despite this stabilization, the formation of the hydrogenated products is energetically favorable, with the overall processes being thermodynamically driven.

## Discussion

The proposed mechanisms revealed significant energy differences between the possible reaction pathways. The primary differences were observed between the potential hydrogenations leading to the final alcohol products and the CO bond cleavage that fosters a chain-growing mechanism. While the computed barriers provide valuable information on how the reaction may proceed and which pathways are most feasible, we further complement these data with kinetic calculations. These calculations allow us to evaluate the possibility of tunnelling and to estimate an approximate temperature based on the calculated reaction rate constants.

As previously mentioned, we employed an RRKM scheme to compute the reaction rate constants, in which tunnelling effects were included because of the low temperatures and participation of H atoms. Calculated rate constants were used to construct Arrhenius plots (available in Figures S1–S5 in the Supporting Information). For a better comparison and contextualization of the FTT processes within the ISM framework, a summary of the temperatures and reaction rate constants is also presented in Table 1. Note that the computed constants are presented in both seconds^–1^ (s^–1^, Earth standards) and years^–1^ (yr^–1^, astronomical standards). It is worth mentioning that within an astrophysical context, a rate constant of 1 yr^–1^ is considered the lower limit at which a given reaction is relatively fast according to astronomical time scales, taking the age of a molecular cloud as 10^6^ yr. Thus, in our kinetic analysis, we determined at which temperature the elementary steps have a *k* = 1 yr^–1^. Moreover, we have also calculated the rate constant considering tunnelling, as tunnelling effects are relevant in the coldest regions of the interstellar medium to explain the formation of some astrochemical complex organic molecules.^85^

**Table 1:** Calculated Temperatures (*T*) and Rate Constants (*k*) Both in years^–1^ (yr^–1^) and seconds^–1^ (s^–1^) of all the Elementary Steps Involved in the First CO Insertion, Second CO Insertion, Methanation, and CH_2_CH_2_ and CH_3_CH_3_ Formation Mechanisms^a^

mechanism	system	*T* (K)	*k* (yr^–1^)	*T* (K)	*k* (s^–1^)	Δ*U* ^‡^	Δ*U*_Rx_
First CO-Insertion							
hydrogenations	TS[HCO]	100 (99)	1.59 (1.47)	154 (153)	1.06 (1.03)	9.3	–9.3
	TS[H_2_CO]	403	1.10	654	1.01	36.6	8.9
	TS[H_3_CO]	377 (374)	1.03 (1.07)	593 (589)	1.00 (1.01)	34.7	–31.4
	TS[H_3_COH]	343 (335)	1.05 (1.07)	514 (505)	1.04 (1.01)	33.6	–12.9
							
C–O dissociations	TS[C + O]	886	1.05	>1000^b^	1.00^b^	90.3	–164.8
	TS[HC + O]	213 (211)	1.03 (1.07)	325 (322)	1.10 (1.05)	20.5	–57.5
	TS[H_2_C + O]	99 (95)	1.50 (1.44)	151 (148)	1.01 (1.07)	9.3	–35.8
	TS[H_3_C + O]	173 (169)	1.13 (1.06)	261 (259)	1.01 (1.05)	16.9	–57.2
Second CO-Insertion							
hydrogenations	TS[CH_2_CO]	386 (384)	1.08 (1.10)	607 (604)	1.04 (1.05)	36.7	–7.2
	TS[CH_2_CHO]	69 (8)	1.50 (1.01)	114 (56)	1.33 (1.03)	6.5	–18.8
	TS[CH_3_CHO]	431	1.10	674	1.00	40.2	17.0
	TS[CH_3_CH_2_O]	546	1.06	842	1.02	52.3	8.3
	TS[CH_3_CH_2_OH]	309	1.02	470	1.02	29.9	13.2
C–O dissociations	TS[CH_2_C + O]	567 (566)	1.07 (1.07)	881 (880)	1.02 (1.03)	53.7	–38.6
	TS[CH_2_CH + O]	246 (244)	1.11 (1.09)	398 (395)	1.05 (1.02)	22.5	–88.2
	TS[CH_3_CH + O]	267 (264)	1.13 (1.08)	416 (412)	1.07 (1.05)	25.3	–14.5
	TS[CH_3_CH_2_ + O]	191 (187)	1.20 (1.03)	288 (285)	1.12 (1.03)	18.6	–92.3
methanation	TS[CH_3_]	586 (583)	1.05 (1.00)	892 (888)	1.03 (1.01)	58.4	–24.6
	TS[CH_4_]	323 (321)	1.13 (1.01)	472 (470)	1.07 (1.04)	33.8	–16.1
CH_2_CH_2_ formation	TS[CH_2_CH + H]	370 (368)	1.04 (1.01)	584 (581)	1.04 (1.01)	31.4	–10.2
CH_3_CH_3_ formation	TS[CH_3_CH_2_ + H]	355 (361)	1.13 (1.00)	545 (551)	1.05 (1.05)	35.0	–23.1

a Values in parentheses are rate constats considering tunnelling. The ZPE-corrected energy barriers (Δ*U* ^‡^) and reaction energies (Δ*U*_Rx_) of each reaction step are also reported.

b It has not been possible to report the exact value of the temperature required as the calculated rate constants are limited to 1000 K.

In the first CO insertion mechanism, it has been reported that the initial hydrogenation leading to [HCO] has a small barrier of only 9.3 kcal mol^–1^, while the subsequent hydrogenations have barriers above 30 kcal mol^–1^. The computed rate constants for the hydrogenation steps show that for the first hydrogenation to [HCO], a rate constant of *k* ≈ 1 yr^–1^ is already reached at 100 K (and *k* ≈ 1 s^–1^ at 154 K). For the following hydrogenations, a rate constant of *k* ≈ 1 yr^–1^ is reached at temperatures above 300 K (above 500 K for *k* ≈ 1 s^–1^). On the other hand, most C–O dissociation barriers are smaller, except for the direct CO cleavage, which has an extremely high barrier of 90.3 kcal mol^–1^. For this initial reaction step, a rate constant of *k* ≈ 1 yr^–1^ is achieved at 886 K. However, the other dissociation barriers are significantly lower, with computed constants reaching *k* ≈ 1 yr^–1^ at temperatures about 100 K (150 K for *k* ≈ 1 s^–1^). The kinetic data predict thus that, in the coldest regions, dissociation mechanisms prevail over subsequent hydrogenations, making the dissociated products the primary products within this first CO insertion mechanism.

For the second CO insertion mechanism, the first required step is the direct CH_2_–CO coupling forming ketene. The computed rate constant of *k* ≈ 1 yr^–1^ for this reaction step is reached at 386 K (*k* ≈ 1 s^–1^ at 607 K). The successive hydrogenations of ketene leading to the formation of ethanol behave similarly to the methanol reaction pathway. The first hydrogenation of the intermediate ketene has a small barrier of 6.5 kcal mol^–1^, comparable to the initial CO hydrogenation in the methanol mechanism. This step reaches a rate constant of *k* ≈ 1 yr^–1^ at 69 K (*k* ≈ 1 s^–1^ at 114 K). The other subsequent hydrogenations are relatively higher in energy, with *k* ≈ 1 yr^–1^ achieved at temperatures above 300 K (above 450 K for *k* ≈ 1 s^–1^). The possible dissociation mechanisms behave similar to those of the first CO insertion mechanism. The direct cleavage of the CO bond in the ketene intermediate [CH_2_C + O] has a high barrier, with a rate constant of *k* ≈ 1 yr^–1^ at 567 K (*k* ≈ 1 s^–1^ at 881 K). However, hydrogenation of ketene significantly reduces the CO dissociation barriers, with further CO bond cleavages showing *k* ≈ 1 yr^–1^ at temperatures already above 150 K.

The kinetic data predict that, in cold regions, ketene can be relatively formed and be hydrogenated once, but after that, dissociation reactions prevail. Note that tunnelling effects are not relevant in these processes, and when present, they only reduce the temperature by a few Kelvins to reach a rate constant equal to 1 year^–1^. When they gain importance (namely, at the very low temperatures) the calculated rate constants are almost null, such that kinetics can be described classically.

Regarding the methanation mechanism, the reported barriers are substantially higher in energy, although both reported steps are exergonic. Tunnelling is also not relevant in either of these steps. The computed rate constants show that the first hydrogenation of CH_2_ has a *k* ≈ 1 yr^–1^ at a temperature of 586 K (*k* ≈ 1 s^–1^ at 892 K), while the second hydrogenation (presenting a smaller barrier) a rate constant of *k* ≈ 1 yr^–1^ is reached at 323 K (*k* ≈ 1 s^–1^ at 472 K). Thus, in a context of low temperatures, the direct hydrogenation of the CH_2_ intermediate is not energetically favorable compared to the CH_2_–CO coupling. Finally, the hydrogenations to form CH_2_CH_2_ and CH_3_CH_3_ show relatively similar barriers, both around the 30 kcal mol^–1^, and therefore constants *k* ≈ 1 yr^–1^ are reached at temperatures beyond the 350 K (*k* ≈ 1 s^–1^ beyond the 500 K).

Based on the obtained kinetic data, the most efficient pathway in the first CO—insertion scenario begins with the hydrogenation of CO, followed by the cleavage of the C–O bond. In all cases, the subsequent hydrogenation steps leading to methanol synthesis require higher temperatures than the corresponding C–O dissociation steps. A similar trend is observed in the second CO-insertion: after CH_2_–CO coupling to form ketene and its initial hydrogenation, C–O bond cleavage remains less temperature-demanding than the final formation of ethanol. Lastly, methanation processes are found to be more energy-intensive than CH_2_–CO coupling.

The formation of these radicals results from the predominance of the chain-growth mechanism over alcohol formation. Chain growth leads to the production of hydrocarbon chains, which is particularly relevant given that hydrocarbons (alkanes and alkenes) are widely detected not only in the interstellar medium but also on exoplanets and in meteoritic samples. Our work proposes a novel reaction pathway for the formation of such iCOMs in environments where traditional gas—grain chemistry falls short. This represents a significant advancement in our understanding of the potential formation mechanisms of these molecules in space, offering a plausible explanation for their presence across diverse astrophysical environments.

Previous studies have reported the catalytic efficiency of Fe single-atom catalysts supported on SiO_2_ for FTT reactions under ISM conditions, revealing a competition between methanol formation and the chain-growth mechanism.^40,41^ In contrast, our Fe_13_@SiO_2_ system not only exhibits higher reactivity, evidenced by lower activation barriers and reduced temperature requirements, but also demonstrates a pronounced preference for the chain-growth mechanism. This shift arises because the dissociation of hydrogenated CO intermediates requires less energy than full hydrogenation to methanol, making chain elongation the dominant pathway.

These findings are consistent with the work of Martinez-Bachs et al., who demonstrated that methanol formation is highly limited in FTT reactions on FeS surfaces.^39^ Additionally, our results align with terrestrial FTT studies, which report that direct CO cleavage is energetically restricted and that hydrogenation is essential for weakening the C–O bond. However, a key difference arises in the final product distribution: while terrestrial FTT (under high H_2_ coverage) favors methanation, our astrochemical conditions (low H_2_ coverage) suppress this pathway, instead promoting hydrocarbon chain growth.^78–83^ This discrepancy highlights how reaction environment (e.g., H_2_ availability and temperature) critically influences product selectivity in FTT chemistry.

Kinetic data helps us identify which stages of planetary formation these reaction processes are feasible based on the temperature requirements to overcome the reported barriers. In all cases, a minimum temperature of at least 100 K is necessary, making these reaction processes impossible in the coldest stages like dense molecular clouds. However, warmer regions like protostellar envelopes, protoplanetary disks and protoplanetary environments are proper scenarios for FTT reactions to occur.

These environments are complex regions where different physical phenomena coexist, yet they contain a region where grains are present and exposed to the gas phase, opening the path to heterogeneous catalysis.^86^ Moreover, such regions are denser than interstellar clouds (atomic densities >10^7^ cm^–3^) and rich in CO and H_2_.^87,88^ Thus, the FTT reactions reported in this work would operate in these regions, first due to the presence of catalytic material and second due to the abundance of the necessary reactants.

Remarkably, such regions are extraordinarily rich in various carbon-chain molecules. Therefore, our results align with these observations, as the main products of the FTT reactions catalyzed by Fe_13_@SiO_2_ arise from the dissociation of activated CO, giving rise to the highly stabilized radicals HC, H_2_C, H_3_C, CH_2_CH, CH_3_CH, and CH_3_CH_2_. These radicals can either undergo hydrogenation to form the relevant hydrocarbons or interact with other radical species present in the environment, leading to the formation of products such as CH_3_CH_2_CN.

In this context and based on our calculations, FTT reactions in the interstellar medium may not primarily contribute to the final synthesis of a specific product. Instead, they play a pivotal role in activating CO and promoting the initial hydrogenation, which then is essential for the posterior cleavage of the CO bond. Moreover, these results align with experimental outputs, which observed the formation of short-chain hydrocarbons rather than alcohol.^35–38^ This process facilitates the subsequent formation of C_*x*_H_*y*_ radical species that can play significant roles in the broader picture of astrochemistry within the complex star- and planetary-forming environments. In this sense, the occurrence of FTT reactions can explain the presence of these species in places where ice chemistry (dominated by cold temperatures and atomic H additions) is not operative, therefore, in relatively warmer regions where ices have sublimated, and the surfaces of bare dust grains are exposed to the gas phase. This would indeed be the case of alcohols, alkanes and alkenes identified in hot cores/corinos (the most central regions close to the protostars),^89^ in the innermost regions of the protoplanetary disks,^90^ and in the primitive atmospheres of terrestrial-like planets and/or planetary moons.^91^ Additionally, FTT processes can also explain over abundances of certain molecules in certain regions that cannot be explained by current astrochemical models, like methanol in shock regions, outflows and jets.^92,93^

## Conclusions

In this study, we have comprehensively evaluated the catalytic performance of the Fe_13_ nanocluster supported on a SiO_2_ surface (Fe_13_@SiO_2_) catalyst for Fischer–Tropsch-type (FTT) synthesis of alcohols (CH_3_OH and CH_3_CHOH), alkanes (CH_4_ and CH_3_CH_3_) and alkenes (CH_2_CH_2_) to assess its occurrence in outer space. In addition to providing a mechanistic study, kinetic analysis based on RRKM theory was performed to predict the feasibility of heterogeneous catalysis in different astrophysical environments. In all cases, the reaction mechanism was investigated using the minimal surface coverage (i.e., considering only one molecule of H_2_ and CO at a time), in consistency with the low densities available in target environments.

Our DFT calculations indicate that after the initial hydrogenation of stable species CO and ketene, the chain-growth mechanism prevails over alcohol formation in both the first and second CO—insertion scenarios. This is because subsequent hydrogenation steps exhibit higher energy barriers than the corresponding CO dissociation steps. Additionally, methanation via CH_2_–H_2_ coupling presents higher barriers than the chain-growth pathway following CH_2_–CO coupling, further reinforcing the preference for molecular growth over methane formation.

The reported kinetic data further support the idea that, in mild-temperature environments, the dissociation mechanism is favored over alcohol formation. This suggests that Fe_13_@SiO_2_-catalyzed FTT reactions are feasible in regions where temperatures are at least above 100 K. Protostellar environments, protoplanetary disk regions, and planetary environments are suitable scenarios satisfying these conditions due to their varying temperatures and the presence of both the catalyst and significant amounts of H_2_ and CO. Our results point out that the occurrence of FTT can explain the presence of some of its products in regions in which ice chemistry cannot justify, that is, warmer regions than the cold interstellar clouds in which ices have sublimated.

Overall, heterogeneous (astro)­catalytic processes based on FTT reactions are relevant in astrochemistry, not necessarily in the direct synthesis of specific molecules, but as mechanisms to activate CO, promote the initial hydrogenation, and subsequently enable CO bond cleavages. This activation results in the formation of various C_*x*_H_*y*_ radical species, contributing to the chemical complexity of astrochemical environments.

## Supplementary Material



## Data Availability

The computational data sets presented in this study is open sourced at the CORA database (10.34810/data2028). In the online repository are collected, the SiO_2_ slab in CIF format, all the geometries in XYZ format, the vibration outputs, examples of the different inputs and outputs and all the kinetic values.

## Notes

The Supporting Information includes the data obtained from the benchmark study and all the computational data including all the relative energies, absolute energies in Hartree, and relative barriers and thermodynamics at both levels of theory (PBEsol and B3LYP) as well as with zero-point and Gibbs energy corrections (PDF)
