# Supplementary material for: Comprehensive Atomistic Simulations of Fischer–Tropsch in Outer Space: Astrocatalysis by Fe13–Supported Nanoclusters on SiO2
Source: J Phys Chem C Nanomater Interfaces. 2025 May 20;129(22):10069–82. doi: 10.1021/acs.jpcc.5c01472 (PMC12147206; doi:10.1021/acs.jpcc.5c01472)
Supplement: Supplementary file 1 [file jp5c01472_si_001.pdf]

# Supporting Information

# Comprehensive Atomistic Simulations of Fischer-Tropsch in Outer Space: Astrocatalysis by $Fe_{13}$-Supported Nanoclusters on $SiO_2$

*Gerard Pareras,[a*] Victoria Cabedo,[b] Martin McCoustra,[b] and Albert Rimola[a*]*

[a]Departament de Química, Universitat Autònoma de Barcelona, 08193 Bellaterra, Catalonia, Spain

[b]Institute of Chemical Sciences, School of Engineering and Physical Sciences, Heriot-Watt University, Edinburgh, EH14 4AS, Scotland

[*]Corresponding authors: gerard.pareras@uab.cat, albert.rimola@uab.cat

**Table of contents**

1. Benchmarking study ........................................ 3

2. Summary of all the computational data ........................................ 3

2.1. First CO-insertion mechanism ........................................ 3

2.2. Formation of $H_2O$ ........................................ 7

2.3. $CH_2$ from CH ........................................ 7

2.4. Second CO-insertion mechanism ........................................ 8

2.5. Methanation ........................................ 12

2.6. Ethylene formation. ........................................ 12

2.7. Ethane formation ........................................ 14

2.8. Relative barriers and thermodynamics ........................................ 15

3. Kinetic data ........................................ 16

3.1. Arrhenius plots for the CO – first insertion path. ........................................ 16

3.2. Arrhenius plots second CO – insertion. ........................................ 18

3.3. Arrhenius plots methanation ........................................ 20

3.4. Arrhenius plots ethylene and ethane formation ........................................ 20

# 1. Benchmarking study

**Table S1.** Relative energies (Rel. E., in kcal mol$^{-1}$) for the different electronic states of the $Fe_{07}$ nanocluster at different levels of theory. The relative error (Rel. Err.) with respect to CCSDT (in %), is for the total energy of the system and divided per atom. The absolute energy (Abs. E.) for each electronic state at each level of theory are in Hartree.

| QM method | Rel. E. | | Rel. Err. (%) | | Abs. E. | |
|---|---|---|---|---|---|---|
| | Triplet | Singlet | Total | Per atom | Triplet | Singlet |
| CCSDT | 0.0 | 205.8 | 0.00 | 0.0 | -7574.74 | -7574.42 |
| PBE-D3(BJ) | 0.0 | 148.7 | -27.7 | -4.6 | -7580.98 | -7580.74 |
| PBE0-D3(BJ) | 0.0 | 306.3 | 48.8 | 8.1 | -7580.77 | -7580.28 |
| B3LYP-D3(BJ) | 0.0 | 312.4 | 51.8 | 8.6 | -7581.83 | -7581.34 |
| BHLYP-D3(BJ) | 0.0 | 424.1 | 106.1 | 17.7 | -7581.91 | -7581.23 |
| wB97X-D3(BJ) | 0.0 | 332.0 | 61.4 | 10.2 | -7582.28 | -7581.75 |

# 2. Summary of all the computational data

## 2.1. First CO-insertion mechanism

**Table S2.** Relative energies (in kcal mol$^{-1}$) for the methanol formation reaction pathway: relative potential energies (ΔE) at the PBEsol-D3(BJ) and B3LYP-D3(BJ)//PBEsol-D3(BJ)levels of theory, relative zero-point energy-corrected energies (ΔU) and Gibbs energies (ΔG) at 200, 400 and 600 K, calculated at the B3LYP-D3(BJ)//PBEsol-D3(BJ) level of theory.

| System | PBEsol-D3(BJ) | B3LYP-D3(BJ)//PBEsol-D3(BJ) | | | | |
|---|---|---|---|---|---|---|
| | ΔE | ΔE | ΔU | $\Delta G_{(200)}$ | $\Delta G_{(400)}$ | $\Delta G_{(600)}$ |
| $Fe_{13}@SiO_2$ | 0.00 | 0.0 | 0.0 | 0.0 | 0.0 | 0.0 |
| [$H_2$] | -17.55 | -16.4 | -13.0 | -12.0 | -10.0 | -8.0 |
| [$H_2$+CO] | -70.75 | -22.2 | -18.9 | -17.7 | -15.8 | -14.5 |
| TS[HCO] | -39.16 | -11.8 | -9.6 | -9.0 | -8.3 | -8.1 |
| [HCO+H] | -48.09 | -33.4 | -28.2 | -27.4 | -26.1 | -25.4 |
| TS[$H_2CO$] | -28.78 | 4.1 | 8.4 | 9.0 | 10.0 | 10.8 |
| [$H_2CO$] | -44.06 | -26.6 | -19.3 | -18.2 | -16.5 | -15.3 |
| [$H_2CO+H_2$] | -68.25 | -30.9 | -23.0 | -22.0 | -20.1 | -18.9 |
| TS[$H_3CO$+H] | -50.11 | 3.2 | 11.7 | 13.5 | 16.5 | 19.0 |
| [$H_3CO$+H] | -84.51 | -67.5 | -54.4 | -53.0 | -50.6 | -48.5 |
| TS[$H_3COH$] | -42.87 | -30.7 | -20.8 | -19.8 | -18.5 | -17.5 |
| [$H_3COH$] | -75.02 | -82.9 | -67.3 | -65.9 | -63.6 | -61.8 |

**Table S3.** Absolute energies (in Hartree) for the methanol formation reaction pathway, including potential energies (E) at the PBEsol-D3(BJ) and B3LYP-D3(BJ)//PBEsol-D3(BJ) levels of theory, zero-point energy-corrected energies (U) and Gibbs energies (G) at 200, 400 and 600 K at the B3LYP-D3(BJ)//PBEsol-D3(BJ) level of theory.

| System | PBEsol-D3(BJ) | B3LYP-D3(BJ)//PBEsol-D3(BJ) | | | | |
|---|---|---|---|---|---|---|
| | E | E | U | $G_{(200)}$ | $G_{(400)}$ | $G_{(600)}$ |
| $Fe_{13}@SiO_2$ | -3811.32 | -3806.30 | -3806.28 | -3806.29 | -3806.31 | -3806.35 |
| [$H_2$] | -3812.52 | -3807.50 | -3807.46 | -3807.47 | -3807.49 | -3807.53 |
| [$H_2$+CO] | -3834.30 | -3829.15 | -3829.11 | -3829.11 | -3829.13 | -3829.17 |

| | | | | | | |
|---|---|---|---|---|---|---|
| TS[HCO] | -3834.25 | -3829.13 | -3829.09 | -3829.10 | -3829.12 | -3829.16 |
| [HCO+H] | -3834.27 | -3829.16 | -3829.12 | -3829.13 | -3829.15 | -3829.19 |
| TS[$H_2$CO] | -3834.24 | -3829.10 | -3829.06 | -3829.07 | -3829.09 | -3829.13 |
| [$H_2$CO] | -3834.26 | -3829.15 | -3829.11 | -3829.11 | -3829.14 | -3829.17 |
| [$H_2$CO+$H_2$] | -3835.47 | -3830.33 | -3830.27 | -3830.28 | -3830.30 | -3830.35 |
| TS[$H_3$CO+H] | -3835.44 | -3830.27 | -3830.21 | -3830.22 | -3830.25 | -3830.29 |
| [$H_3$CO+H] | -3835.50 | -3830.39 | -3830.32 | -3830.33 | -3830.35 | -3830.39 |
| TS[$H_3$COH] | -3835.43 | -3830.33 | -3830.27 | -3830.27 | -3830.30 | -3830.34 |
| [$H_3$COH] | -3835.48 | -3830.41 | -3830.34 | -3830.35 | -3830.37 | -3830.41 |

**Table S4.** Collected the relative energies in kcal mol$^{-1}$ for the C-O dissociation reaction pathway, including the relative electronic energies at the PBEsol-D3(BJ) and B3LYP-D3(BJ)//PBEsol-D3(BJ) levels of theory, and the relative internal energies and Gibbs energies at the 200, 400 and 600 K at the B3LYP-D3(BJ)//PBEsol-D3(BJ) level of theory.

| System | PBEsol-D3(BJ) | B3LYP-D3(BJ)//PBEsol-D3(BJ) | | | | |
|---|---|---|---|---|---|---|
| | ΔE | ΔE | ΔU | $\Delta G_{(200)}$ | $\Delta G_{(400)}$ | $\Delta G_{(600)}$ |
| $Fe_{13}$@$SiO_2$ | 0.00 | 0.0 | 0.0 | 0.0 | 0.0 | 0.0 |
| [$H_2$] | -17.55 | -16.4 | -13.0 | -12.0 | -10.0 | -8.0 |
| [$H_2$+CO] | -70.75 | -22.2 | -18.9 | -17.7 | -15.8 | -14.5 |
| TS[C+O] | 17.81 | 71.2 | 71.3 | 70.3 | 68.9 | 67.0 |
| [C+O] | -82.3 | -95.8 | -93.4 | -92.7 | -91.8 | -91.4 |

**Table S5.** Collected the absolute energies in Hartree for the for the C-O dissociation reaction pathway, including electronic energies at the PBEsol-D3(BJ) and B3LYP-D3(BJ)//PBEsol-D3(BJ) levels of theory, and the internal energies and Gibbs energies at the 200, 400 and 600 K at the B3LYP-D3(BJ)//PBEsol-D3(BJ)level of theory.

| System | PBEsol-D3(BJ) | B3LYP-D3(BJ)//PBEsol-D3(BJ) | | | | |
|---|---|---|---|---|---|---|
| | E | E | U | $G_{(200)}$ | $G_{(400)}$ | $G_{(600)}$ |
| $Fe_{13}$@$SiO_2$ | -3811.32 | -3806.30 | -3806.28 | -3806.29 | -3806.31 | -3806.35 |
| [$H_2$] | -3812.52 | -3807.50 | -3807.46 | -3807.47 | -3807.49 | -3807.53 |
| [$H_2$+CO] | -3834.30 | -3829.15 | -3829.11 | -3829.11 | -3829.13 | -3829.17 |
| TS[C+O] | -3834.16 | -3829.00 | -3828.96 | -3828.97 | -3829.00 | -3829.04 |
| [C+O] | -3834.32 | -3829.26 | -3829.22 | -3829.23 | -3829.26 | -3829.29 |

**Table S6.** Collected the relative energies in kcal mol$^{-1}$ for the HC-O dissociation reaction pathway, including the relative electronic energies at the PBEsol-D3(BJ) and B3LYP-D3(BJ)//PBEsol-D3(BJ) levels of theory, and the relative internal energies and Gibbs energies at the 200, 400 and 600 K at the B3LYP-D3(BJ)//PBEsol-D3(BJ) level of theory.

| System | PBEsol-D3(BJ) | B3LYP-D3(BJ)//PBEsol-D3(BJ) | | | | |
|---|---|---|---|---|---|---|
| | ΔE | ΔE | ΔU | $\Delta G_{(200)}$ | $\Delta G_{(400)}$ | $\Delta G_{(600)}$ |
| $Fe_{13}$@$SiO_2$ | 0.00 | 0.0 | 0.0 | 0.0 | 0.0 | 0.0 |
| [$H_2$] | -17.55 | -16.4 | -13.0 | -12.0 | -10.0 | -8.0 |
| [$H_2$+CO] | -70.75 | -22.2 | -18.9 | -17.7 | -15.8 | -14.5 |
| TS[HCO] | -39.16 | -11.8 | -9.6 | -9.0 | -8.3 | -8.1 |
| [HCO+H] | -48.09 | -33.4 | -28.2 | -27.4 | -26.1 | -25.4 |
| TS[HC+O] | -32.72 | -9.7 | -7.6 | -7.5 | -7.4 | -7.9 |
| [HC+O] | -75.75 | -89.7 | -85.6 | -84.5 | -82.8 | -81.7 |

**Table S7.** Collected the absolute energies in Hartree for the for the HC-O dissociation reaction pathway, including electronic energies at the PBEsol-D3(BJ) and B3LYP-D3(BJ)//PBEsol-D3(BJ) levels of theory, and the internal energies and Gibbs energies at the 200, 400 and 600 K at the B3LYP-D3(BJ)//PBEsol-D3(BJ) level of theory.

| System | PBEsol-D3(BJ) | B3LYP-D3(BJ)//PBEsol-D3(BJ) | | | | |
|---|---|---|---|---|---|---|
| | E | E | U | $G_{(200)}$ | $G_{(400)}$ | $G_{(600)}$ |
| $Fe_{13}@SiO_2$ | -3811.32 | -3806.30 | -3806.28 | -3806.29 | -3806.31 | -3806.35 |
| [$H_2$] | -3812.52 | -3807.50 | -3807.46 | -3807.47 | -3807.49 | -3807.53 |
| [$H_2$+CO] | -3834.30 | -3829.15 | -3829.11 | -3829.11 | -3829.13 | -3829.17 |
| TS[HCO] | -3834.25 | -3829.13 | -3829.09 | -3829.10 | -3829.12 | -3829.16 |
| [HCO+H] | -3834.27 | -3829.16 | -3829.12 | -3829.13 | -3829.15 | -3829.19 |
| TS[HC+O] | -3834.24 | -3829.13 | -3829.09 | -3829.09 | -3829.12 | -3829.16 |
| [HC+O] | -3834.31 | -3829.25 | -3829.21 | -3829.22 | -3829.24 | -3829.28 |

**Table S8.** Collected the relative energies in kcal mol$^{-1}$ for the $H_2$C-O dissociation reaction pathway, including the relative electronic energies at the PBEsol-D3(BJ) and B3LYP-D3(BJ)//PBEsol-D3(BJ) levels of theory, and the relative internal energies and Gibbs energies at the 200, 400 and 600 K at the B3LYP-D3(BJ)//PBEsol-D3(BJ) level of theory.

| System | PBEsol-D3(BJ) | B3LYP-D3(BJ)//PBEsol-D3(BJ) | | | | |
|---|---|---|---|---|---|---|
| | ΔE | ΔE | ΔU | $\Delta G_{(200)}$ | $\Delta G_{(400)}$ | $\Delta G_{(600)}$ |
| $Fe_{13}@SiO_2$ | 0.00 | 0.0 | 0.0 | 0.0 | 0.0 | 0.0 |
| [$H_2$] | -17.55 | -16.4 | -13.0 | -12.0 | -10.0 | -8.0 |
| [$H_2$+CO] | -70.75 | -22.2 | -18.9 | -17.7 | -15.8 | -14.5 |
| TS[HCO] | -39.16 | -11.8 | -9.6 | -9.0 | -8.3 | -8.1 |
| [HCO+H] | -48.09 | -33.4 | -28.2 | -27.4 | -26.1 | -25.4 |
| TS[$H_2$CO] | -28.78 | 4.1 | 8.4 | 9.0 | 10.0 | 10.8 |
| [$H_2$CO] | -44.06 | -26.6 | -19.3 | -18.2 | -16.5 | -15.3 |
| TS[$H_2$C+O] | -36.44 | -15.3 | -10.1 | -9.5 | -8.7 | -8.3 |
| [$H_2$C+O] | -75.72 | -61.5 | -55.2 | -54.2 | -52.7 | -51.7 |

**Table S9.** Collected the absolute energies in Hartree for the for the $H_2$C-O dissociation reaction pathway, including electronic energies at the PBEsol-D3(BJ) and B3LYP-D3(BJ)//PBEsol-D3(BJ) levels of theory, and the internal energies and Gibbs energies at the 200, 400 and 600 K at the B3LYP-D3(BJ)//PBEsol-D3(BJ) level of theory.

| System | PBEsol-D3(BJ) | B3LYP-D3(BJ)//PBEsol-D3(BJ) | | | | |
|---|---|---|---|---|---|---|
| | E | E | U | $G_{(200)}$ | $G_{(400)}$ | $G_{(600)}$ |
| $Fe_{13}@SiO_2$ | -3811.32 | -3806.30 | -3806.28 | -3806.29 | -3806.31 | -3806.35 |
| [$H_2$] | -3812.52 | -3807.50 | -3807.46 | -3807.47 | -3807.49 | -3807.53 |
| [$H_2$+CO] | -3834.30 | -3829.15 | -3829.11 | -3829.11 | -3829.13 | -3829.17 |
| TS[HCO] | -3834.25 | -3829.13 | -3829.09 | -3829.10 | -3829.12 | -3829.16 |
| [HCO+H] | -3834.27 | -3829.16 | -3829.12 | -3829.13 | -3829.15 | -3829.19 |
| TS[$H_2$CO] | -3834.24 | -3829.10 | -3829.06 | -3829.07 | -3829.09 | -3829.13 |
| [$H_2$CO] | -3834.26 | -3829.15 | -3829.11 | -3829.11 | -3829.14 | -3829.17 |
| TS[$H_2$C+O] | -3834.25 | -3829.13 | -3829.09 | -3829.10 | -3829.12 | -3829.16 |
| [$H_2$C+O] | -3834.31 | -3829.21 | -3829.16 | -3829.17 | -3829.19 | -3829.23 |

**Table S10.** Collected the relative energies in kcal mol$^{-1}$ for the $H_3$C-O dissociation reaction pathway, including the relative electronic energies at the PBEsol-D3(BJ) and B3LYP-D3(BJ)//PBEsol-D3(BJ) levels of theory, and the relative internal energies and Gibbs energies at the 200, 400 and 600 K at the B3LYP-D3(BJ)//PBEsol-D3(BJ) level of theory.

| System | PBEsol-D3(BJ) | B3LYP-D3(BJ)//PBEsol-D3(BJ) |
|---|---|---|

| | ΔE | ΔE | ΔU | $\Delta G_{(200)}$ | $\Delta G_{(400)}$ | $\Delta G_{(600)}$ |
|---|---|---|---|---|---|---|
| $Fe_{13}$@$SiO_2$ | 0.00 | 0.0 | 0.0 | 0.0 | 0.0 | 0.0 |
| [$H_2$] | -17.55 | -16.4 | -13.0 | -12.0 | -10.0 | -8.0 |
| [$H_2$+CO] | -70.75 | -22.2 | -18.9 | -17.7 | -15.8 | -14.5 |
| TS[HCO] | -39.16 | -11.8 | -9.6 | -9.0 | -8.3 | -8.1 |
| [HCO+H] | -48.09 | -33.4 | -28.2 | -27.4 | -26.1 | -25.4 |
| TS[$H_2$CO] | -28.78 | 4.1 | 8.4 | 9.0 | 10.0 | 10.8 |
| [$H_2$CO] | -44.06 | -26.6 | -19.3 | -18.2 | -16.5 | -15.3 |
| [$H_2$CO+$H_2$] | -68.25 | -30.9 | -23.0 | -22.0 | -20.1 | -18.9 |
| TS[$H_3$CO+H] | -50.11 | 3.2 | 11.7 | 13.5 | 16.5 | 19.0 |
| [$H_3$CO+H] | -84.51 | -67.5 | -54.4 | -53.0 | -50.6 | -48.5 |
| TS[$H_3$C+O] | -45.9 | -44.8 | -37.5 | -38.1 | -39.1 | -40.8 |
| [$H_3$C+O] | -142.9 | -130.0 | -111.6 | -109.9 | -105.8 | -101.5 |

**Table S11.** Collected the absolute energies in Hartree for the for the $H_3C$-O dissociation reaction pathway, including electronic energies at the PBEsol-D3(BJ) and B3LYP-D3(BJ)//PBEsol-D3(BJ) levels of theory, and the internal energies and Gibbs energies at the 200, 400 and 600 K at the B3LYP-D3(BJ)//PBEsol-D3(BJ) level of theory.

| System | PBEsol-D3(BJ) | B3LYP-D3(BJ)//PBEsol-D3(BJ) | | | | |
|---|---|---|---|---|---|---|
| | E | E | U | $G_{(200)}$ | $G_{(400)}$ | $G_{(600)}$ |
| $Fe_{13}$@$SiO_2$ | -3811.32 | -3806.30 | -3806.28 | -3806.29 | -3806.31 | -3806.35 |
| [$H_2$] | -3812.52 | -3807.50 | -3807.46 | -3807.47 | -3807.49 | -3807.53 |
| [$H_2$+CO] | -3834.30 | -3829.15 | -3829.11 | -3829.11 | -3829.13 | -3829.17 |
| TS[HCO] | -3834.25 | -3829.13 | -3829.09 | -3829.10 | -3829.12 | -3829.16 |
| [HCO+H] | -3834.27 | -3829.16 | -3829.12 | -3829.13 | -3829.15 | -3829.19 |
| TS[$H_2$CO] | -3834.24 | -3829.10 | -3829.06 | -3829.07 | -3829.09 | -3829.13 |
| [$H_2$CO] | -3834.26 | -3829.15 | -3829.11 | -3829.11 | -3829.14 | -3829.17 |
| [$H_2$CO+$H_2$] | -3835.47 | -3830.33 | -3830.27 | -3830.28 | -3830.30 | -3830.35 |
| TS[$H_3$CO+H] | -3835.44 | -3830.27 | -3830.21 | -3830.22 | -3830.25 | -3830.29 |
| [$H_3$CO+H] | -3835.50 | -3830.39 | -3830.32 | -3830.33 | -3830.35 | -3830.39 |
| TS[$H_3$C+O] | -3835.44 | -3830.35 | -3830.29 | -3830.30 | -3830.33 | -3830.38 |
| [$H_3$C+O] | -3835.59 | -3830.49 | -3830.41 | -3830.42 | -3830.44 | -3830.48 |

## 2.2. Formation of $H_2O$

**Table S12.** Collected the relative energies in kcal mol$^{-1}$ for the formation of $H_2O$, including the relative electronic energies at the PBEsol-D3(BJ) and B3LYP-D3(BJ)//PBEsol-D3(BJ) levels of theory, and the relative internal energies and Gibbs energies at the 200, 400 and 600 K at the B3LYP-D3(BJ)//PBEsol-D3(BJ) level of theory.

| System | PBEsol-D3(BJ) | B3LYP-D3(BJ)//PBEsol-D3(BJ) | | | | |
|---|---|---|---|---|---|---|
| | ΔE | ΔE | ΔU | $\Delta G_{(200)}$ | $\Delta G_{(400)}$ | $\Delta G_{(600)}$ |
| [O+H2] | 0.00 | 0.00 | 0.00 | 0.00 | 0.00 | 0.00 |
| TS[OH+H] | 39.50 | 31.07 | 28.56 | 27.25 | 25.40 | 23.59 |
| [OH+H] | -8.94 | 14.55 | 15.84 | 15.38 | 14.40 | 13.37 |
| TS[H2O] | 19.23 | 30.82 | 28.31 | 27.00 | 25.15 | 23.34 |
| [H2O] | 8.22 | 16.86 | 18.15 | 17.69 | 16.71 | 15.67 |

**Table S13.** Collected the absolute energies in Hartree for the for the formation of $H_2O$, including electronic energies at the PBEsol-D3(BJ) and B3LYP-D3(BJ)//PBEsol-D3(BJ) levels of theory, and the internal energies and Gibbs energies at the 200, 400 and 600 K at the B3LYP-D3(BJ)//PBEsol-D3(BJ) level of theory.

| System | PBEsol-D3(BJ) | B3LYP-D3(BJ)//PBEsol-D3(BJ) | | | | |
|---|---|---|---|---|---|---|
| | E | E | U | $G_{(200)}$ | $G_{(400)}$ | $G_{(600)}$ |
| [O+H2] | -3828.59 | -3856.70 | -3856.64 | -3856.65 | -3856.67 | -3856.71 |
| TS[OH+H] | -3828.53 | -3856.65 | -3856.60 | -3856.60 | -3856.63 | -3856.67 |
| [OH+H] | -3828.61 | -3856.67 | -3856.62 | -3856.62 | -3856.65 | -3856.69 |
| TS[H2O] | -3828.56 | -3856.65 | -3856.60 | -3856.60 | -3856.63 | -3856.67 |
| [H2O] | -3828.58 | -3856.67 | -3856.61 | -3856.62 | -3856.64 | -3856.68 |

## 2.3. $CH_2$ from CH

**Table S14.** Collected the relative energies in kcal mol$^{-1}$ for the formation of $CH_2$ from the hydrogenation of CH, including the relative electronic energies at the PBEsol-D3(BJ) and B3LYP-D3(BJ)//PBEsol-D3(BJ) levels of theory,

and the relative internal energies and Gibbs energies at the 200, 400 and 600 K at the B3LYP-D3(BJ)//PBEsol-D3(BJ) level of theory.

| System | PBEsol-D3(BJ) | B3LYP-D3(BJ)//PBEsol-D3(BJ) | | | | |
|---|---|---|---|---|---|---|
| | ΔE | ΔE | ΔU | $\Delta G_{(200)}$ | $\Delta G_{(400)}$ | $\Delta G_{(600)}$ |
| [CH+H] | 0.0 | 0.00 | 0.00 | 0.00 | 0.00 | 0.00 |
| TS[CH+H] | 14.8 | 15.16 | 12.65 | 11.34 | 9.49 | 7.68 |
| [CH2] | 5.3 | -4.02 | -2.72 | -3.18 | -4.16 | -5.20 |

**Table S15.** Collected the absolute energies in Hartree for the for formation of $CH_2$ from the hydrogenation of CH, including electronic energies at the PBEsol-D3(BJ) and B3LYP-D3(BJ)//PBEsol-D3(BJ) levels of theory, and the internal energies and Gibbs energies at the 200, 400 and 600 K at the B3LYP-D3(BJ)//PBEsol-D3(BJ) level of theory.

| System | PBEsol-D3(BJ) | B3LYP-D3(BJ)//PBEsol-D3(BJ) | | | | |
|---|---|---|---|---|---|---|
| | E | E | U | $G_{(200)}$ | $G_{(400)}$ | $G_{(600)}$ |
| [CH+H] | -3818.21 | -3846.24 | -3846.18 | -3846.18 | -3846.21 | -3846.25 |
| TS[CH+H] | -3818.19 | -3846.21 | -3846.16 | -3846.17 | -3846.19 | -3846.23 |
| [CH2] | -3818.21 | -3846.24 | -3846.18 | -3846.19 | -3846.22 | -3846.25 |

## 2.4. Second CO-insertion mechanism

**Table S16.** Collected the relative energies in kcal $mol^{-1}$ for the ethanol reaction pathway, including the relative electronic energies at the PBEsol-D3(BJ) and B3LYP-D3(BJ)//PBEsol-D3(BJ) levels of theory, and the relative internal energies and Gibbs energies at the 200, 400 and 600 K at the B3LYP-D3(BJ)//PBEsol-D3(BJ) level of theory.

| System | PBEsol-D3(BJ) | B3LYP-D3(BJ)//PBEsol-D3(BJ) | | | | |
|---|---|---|---|---|---|---|
| | ΔE | ΔE | ΔU | $\Delta G_{(200)}$ | $\Delta G_{(400)}$ | $\Delta G_{(600)}$ |
| [$CH_2$] | 0.0 | 0.0 | 0.0 | 0.0 | 0.0 | 0.0 |
| [$CH_2$+CO] | -43.2 | -16.6 | -13.7 | -13.0 | -11.9 | -11.2 |
| TS[$CH_2$CO] | -8.0 | 20.5 | 23.0 | 23.0 | 23.1 | 23.1 |
| [$CH_2$CO] | -41.6 | -26.1 | -20.9 | -20.1 | -18.6 | -17.3 |
| [$CH_2$CO+$H_2$] | -59.3 | -46.5 | -39.5 | -37.9 | -35.2 | -32.8 |
| TS[$CH_2$CHO] | -38.0 | -37.8 | -33.0 | -32.0 | -29.9 | -28.1 |
| [$CH_2$CHO+H] | -71.5 | -67.0 | -58.3 | -57.2 | -55.1 | -53.2 |
| TS[$CH_3$CHO] | -53.0 | -26.3 | -18.1 | -16.7 | -14.2 | -11.8 |
| [$CH_3$CHO] | -63.0 | -52.4 | -41.2 | -39.8 | -37.0 | -34.3 |
| [$CH_3$CHO+$H_2$] | -76.9 | -78.3 | -66.1 | -64.1 | -60.5 | -57.0 |
| TS[CH3$CH_2$O] | -52.0 | -24.8 | -13.7 | -12.0 | -8.7 | -5.5 |
| [CH3$CH_2$O+H] | -81.0 | -73.2 | -57.8 | -56.3 | -53.5 | -50.7 |
| TS[CH3$CH_2$OH] | -51.7 | -42.1 | -27.9 | -27.0 | -25.1 | -23.2 |
| [CH3$CH_2$OH] | -71.7 | -63.8 | -44.6 | -43.0 | -40.1 | -37.3 |

**Table S17.** Collected the absolute energies in Hartree for the for the ethanol reaction pathway, including electronic energies at the PBEsol-D3(BJ) and B3LYP-D3(BJ)//PBEsol-D3(BJ) levels of theory, and the internal energies and Gibbs energies at the 200, 400 and 600 K at the B3LYP-D3(BJ)//PBEsol-D3(BJ) level of theory.

| System | PBEsol-D3(BJ) | B3LYP-D3(BJ)//PBEsol-D3(BJ) | | | | |
|---|---|---|---|---|---|---|
| | E | E | U | $G_{(200)}$ | $G_{(400)}$ | $G_{(600)}$ |
| [$CH_2$] | -3818.21 | -3813.14 | -3813.10 | -3813.10 | -3813.13 | -3813.17 |
| [$CH_2$+CO] | -3839.97 | -3834.80 | -3834.75 | -3834.76 | -3834.78 | -3834.82 |
| TS[$CH_2$CO] | -3839.92 | -3834.74 | -3834.69 | -3834.70 | -3834.73 | -3834.77 |
| [$CH_2$CO] | -3839.97 | -3834.82 | -3834.76 | -3834.77 | -3834.79 | -3834.83 |
| [$CH_2$CO+$H_2$] | -3841.17 | -3836.02 | -3835.95 | -3835.96 | -3835.98 | -3836.03 |
| TS[$CH_2$CHO] | -3841.14 | -3836.00 | -3835.94 | -3835.95 | -3835.98 | -3836.02 |
| [$CH_2$CHO+H] | -3841.19 | -3836.05 | -3835.98 | -3835.99 | -3836.02 | -3836.06 |
| TS[$CH_3$CHO] | -3841.16 | -3835.99 | -3835.92 | -3835.92 | -3835.95 | -3835.99 |
| [$CH_3$CHO] | -3841.18 | -3836.03 | -3835.95 | -3835.96 | -3835.99 | -3836.03 |
| [$CH_3$CHO+$H_2$] | -3842.37 | -3837.24 | -3837.15 | -3837.16 | -3837.19 | -3837.23 |
| TS[CH3$CH_2$O] | -3842.33 | -3837.15 | -3837.07 | -3837.08 | -3837.10 | -3837.15 |
| [CH3$CH_2$O+H] | -3842.38 | -3837.23 | -3837.14 | -3837.15 | -3837.18 | -3837.22 |
| TS[CH3$CH_2$OH] | -3842.33 | -3837.18 | -3837.09 | -3837.10 | -3837.13 | -3837.18 |
| [CH3$CH_2$OH] | -3842.36 | -3837.22 | -3837.12 | -3837.13 | -3837.15 | -3837.20 |

**Table S18.** Collected the relative energies in kcal $mol^{-1}$ for the $CH_2CO$ dissociation reaction pathway, including the relative electronic energies at the PBEsol-D3(BJ) and B3LYP-D3(BJ)//PBEsol-D3(BJ) levels of theory, and the relative internal energies and Gibbs energies at the 200, 400 and 600 K at the B3LYP-D3(BJ)//PBEsol-D3(BJ) level of theory.

| System | PBEsol-D3(BJ) | B3LYP-D3(BJ)//PBEsol-D3(BJ) | | | | |
|---|---|---|---|---|---|---|
| | ΔE | ΔE | ΔU | $\Delta G_{(200)}$ | $\Delta G_{(400)}$ | $\Delta G_{(600)}$ |
| [$CH_2$] | 0.00 | 0.0 | 0.0 | 0.0 | 0.0 | 0.0 |
| [$CH_2$+CO] | -43.19 | -16.6 | -13.7 | -13.0 | -11.9 | -11.2 |
| TS[$CH_2$CO] | -8.04 | 20.5 | 23.0 | 23.0 | 23.1 | 23.1 |
| [$CH_2$CO] | -41.59 | -26.1 | -20.9 | -20.1 | -18.6 | -17.3 |
| TS[$CH_2$C+O] | 3.3 | 29.5 | 32.9 | 33.6 | 34.9 | 36.2 |
| [$CH_2$C+O] | -67.9 | -61.4 | -58.3 | -58.7 | -59.2 | -59.9 |

**Table S19.** Collected the absolute energies in Hartree for the for the $CH_2CO$ dissociation reaction pathway, including electronic energies at the PBEsol-D3(BJ) and B3LYP-D3(BJ)//PBEsol-D3(BJ) levels of theory, and the internal energies and Gibbs energies at the 200, 400 and 600 K at the B3LYP-D3(BJ)//PBEsol-D3(BJ) level of theory.

| System | PBEsol-D3(BJ) | B3LYP-D3(BJ)//PBEsol-D3(BJ) | | | | |
|---|---|---|---|---|---|---|
| | E | E | U | $G_{(200)}$ | $G_{(400)}$ | $G_{(600)}$ |
| [$CH_2$] | -3818.21 | -3813.14 | -3813.10 | -3813.10 | -3813.13 | -3813.17 |
| [$CH_2$+CO] | -3839.97 | -3834.80 | -3834.75 | -3834.76 | -3834.78 | -3834.82 |
| TS[$CH_2$CO] | -3839.92 | -3834.74 | -3834.69 | -3834.70 | -3834.73 | -3834.77 |
| [$CH_2$CO] | -3839.97 | -3834.82 | -3834.76 | -3834.77 | -3834.79 | -3834.83 |
| TS[$CH_2$C+O] | -3839.90 | -3834.73 | -3834.68 | -3834.68 | -3834.71 | -3834.75 |
| [$CH_2$C+O] | -3840.01 | -3834.87 | -3834.82 | -3834.83 | -3834.86 | -3834.90 |

**Table S20.** Collected the relative energies in kcal mol-1 for the $CH_2CHO$ dissociation reaction pathway, including the relative electronic energies at the PBEsol-D3(BJ) and B3LYP-D3(BJ)//PBEsol-D3(BJ) levels of theory, and the relative internal energies and Gibbs energies at the 200, 400 and 600 K at the B3LYP-D3(BJ)//PBEsol-D3(BJ) level of theory.

| System | PBEsol-D3(BJ) | B3LYP-D3(BJ)//PBEsol-D3(BJ) | | | | |
|---|---|---|---|---|---|---|
| | ΔE | ΔE | ΔU | $\Delta G_{(200)}$ | $\Delta G_{(400)}$ | $\Delta G_{(600)}$ |
| [$CH_2$] | 0.00 | 0.0 | 0.0 | 0.0 | 0.0 | 0.0 |
| [$CH_2$+CO] | -43.19 | -16.6 | -13.7 | -13.0 | -11.9 | -11.2 |
| TS[$CH_2$CO] | -8.04 | 20.5 | 23.0 | 23.0 | 23.1 | 23.1 |
| [$CH_2$CO] | -41.59 | -26.1 | -20.9 | -20.1 | -18.6 | -17.3 |
| [$CH_2$CO+$H_2$] | -59.33 | -46.5 | -39.5 | -37.9 | -35.2 | -32.8 |
| TS[$CH_2$CHO] | -38.03 | -37.8 | -33.0 | -32.0 | -29.9 | -28.1 |
| [$CH_2$CHO+H] | -71.51 | -67.0 | -58.3 | -57.2 | -55.1 | -53.2 |
| TS[$CH_2$CH+O] | -27.1 | -43.0 | -35.8 | -34.8 | -32.6 | -30.4 |
| [$CH_2$CH+O] | -107.8 | -151.9 | -146.4 | -146.3 | -146.3 | -146.6 |

**Table S21.** Collected the absolute energies in Hartree for the for the $CH_2CHO$ dissociation pathway, including electronic energies at the PBEsol-D3(BJ) and B3LYP-D3(BJ)//PBEsol-D3(BJ) levels of theory, and the internal energies and Gibbs energies at the 200, 400 and 600 K at the B3LYP-D3(BJ)//PBEsol-D3(BJ) level of theory.

| System | PBEsol-D3(BJ) | B3LYP-D3(BJ)//PBEsol-D3(BJ) | | | | |
|---|---|---|---|---|---|---|
| | E | E | U | $G_{(200)}$ | $G_{(400)}$ | $G_{(600)}$ |
| [$CH_2$] | -3818.21 | -3813.14 | -3813.10 | -3813.10 | -3813.13 | -3813.17 |
| [$CH_2$+CO] | -3839.97 | -3834.80 | -3834.75 | -3834.76 | -3834.78 | -3834.82 |
| TS[$CH_2$CO] | -3839.92 | -3834.74 | -3834.69 | -3834.70 | -3834.73 | -3834.77 |
| [$CH_2$CO] | -3839.97 | -3834.82 | -3834.76 | -3834.77 | -3834.79 | -3834.83 |
| [$CH_2$CO+$H_2$] | -3841.17 | -3836.02 | -3835.95 | -3835.96 | -3835.98 | -3836.03 |
| TS[$CH_2$CHO] | -3841.14 | -3836.00 | -3835.94 | -3835.95 | -3835.98 | -3836.02 |
| [$CH_2$CHO+H] | -3841.19 | -3836.05 | -3835.98 | -3835.99 | -3836.02 | -3836.06 |
| TS[$CH_2$CH+O] | -3841.12 | -3836.01 | -3835.95 | -3835.95 | -3835.98 | -3836.02 |
| [$CH_2$CH+O] | -3841.25 | -3836.19 | -3836.12 | -3836.13 | -3836.16 | -3836.21 |

**Table S22.** Collected the relative energies in kcal mol-1 for the $CH_3CHO$ dissociation reaction pathway, including the relative electronic energies at the PBEsol-D3(BJ) and B3LYP-D3(BJ)//PBEsol-D3(BJ) levels of theory, and the relative internal energies and Gibbs energies at the 200, 400 and 600 K at the B3LYP-D3(BJ)//PBEsol-D3(BJ) level of theory.

| System | PBEsol-D3(BJ) | B3LYP-D3(BJ)//PBEsol-D3(BJ) | | | | |
|---|---|---|---|---|---|---|
| | ΔE | ΔE | ΔU | $\Delta G_{(200)}$ | $\Delta G_{(400)}$ | $\Delta G_{(600)}$ |
| [$CH_2$] | 0.00 | 0.0 | 0.0 | 0.0 | 0.0 | 0.0 |
| [$CH_2$+CO] | -43.19 | -16.6 | -13.7 | -13.0 | -11.9 | -11.2 |
| TS[$CH_2$CO] | -8.04 | 20.5 | 23.0 | 23.0 | 23.1 | 23.1 |
| [$CH_2$CO] | -41.59 | -26.1 | -20.9 | -20.1 | -18.6 | -17.3 |
| [$CH_2$CO+$H_2$] | -59.33 | -46.5 | -39.5 | -37.9 | -35.2 | -32.8 |
| TS[$CH_2$CHO] | -38.03 | -37.8 | -33.0 | -32.0 | -29.9 | -28.1 |
| [$CH_2$CHO+H] | -71.51 | -67.0 | -58.3 | -57.2 | -55.1 | -53.2 |
| TS[$CH_3$CHO] | -53.02 | -26.3 | -18.1 | -16.7 | -14.2 | -11.8 |
| [$CH_3$CHO] | -62.96 | -52.4 | -41.2 | -39.8 | -37.0 | -34.3 |
| TS[$CH_3$CH+O] | -42.2 | -25.0 | -16.0 | -15.3 | -13.7 | -12.1 |
| [$CH_3$CH+O] | -64.9 | -66.7 | -55.7 | -55.0 | -53.3 | -51.7 |

**Table S23.** Collected the absolute energies in Hartree for the for the $CH_3CHO$ dissociation pathway, including electronic energies at the PBEsol-D3(BJ) and B3LYP-D3(BJ)//PBEsol-D3(BJ) levels of theory, and the internal energies and Gibbs energies at the 200, 400 and 600 K at the B3LYP-D3(BJ)//PBEsol-D3(BJ) level of theory.

| System | PBEsol-D3(BJ) | B3LYP-D3(BJ)//PBEsol-D3(BJ) | | | | |
|---|---|---|---|---|---|---|
| | E | E | U | $G_{(200)}$ | $G_{(400)}$ | $G_{(600)}$ |
| [$CH_2$] | -3818.21 | -3813.14 | -3813.10 | -3813.10 | -3813.13 | -3813.17 |
| [$CH_2$+CO] | -3839.97 | -3834.80 | -3834.75 | -3834.76 | -3834.78 | -3834.82 |
| TS[$CH_2$CO] | -3839.92 | -3834.74 | -3834.69 | -3834.70 | -3834.73 | -3834.77 |
| [$CH_2$CO] | -3839.97 | -3834.82 | -3834.76 | -3834.77 | -3834.79 | -3834.83 |
| [$CH_2$CO+$H_2$] | -3841.17 | -3836.02 | -3835.95 | -3835.96 | -3835.98 | -3836.03 |
| TS[$CH_2$CHO] | -3841.14 | -3836.00 | -3835.94 | -3835.95 | -3835.98 | -3836.02 |
| [$CH_2$CHO+H] | -3841.19 | -3836.05 | -3835.98 | -3835.99 | -3836.02 | -3836.06 |
| TS[$CH_3$CHO] | -3841.16 | -3835.99 | -3835.92 | -3835.92 | -3835.95 | -3835.99 |
| [$CH_3$CHO] | -3841.18 | -3836.03 | -3835.95 | -3835.96 | -3835.99 | -3836.03 |
| TS[$CH_3$CH+O] | -3841.14 | -3835.98 | -3835.91 | -3835.92 | -3835.95 | -3835.99 |
| [$CH_3$CH+O] | -3841.18 | -3836.05 | -3835.98 | -3835.99 | -3836.01 | -3836.06 |

**Table S24.** Collected the relative energies in kcal mol$^{-1}$ for the $CH_3CH_2O$ dissociation reaction pathway, including the relative electronic energies at the PBEsol-D3(BJ) and B3LYP-D3(BJ)//PBEsol-D3(BJ) levels of theory, and the relative internal energies and Gibbs energies at the 200, 400 and 600 K at the B3LYP-D3(BJ)//PBEsol-D3(BJ) level of theory.

| System | PBEsol-D3(BJ) | B3LYP-D3(BJ)//PBEsol-D3(BJ) | | | | |
|---|---|---|---|---|---|---|
| | ΔE | ΔE | ΔU | $\Delta G_{(200)}$ | $\Delta G_{(400)}$ | $\Delta G_{(600)}$ |
| [$CH_2$] | 0.0 | 0.0 | 0.0 | 0.0 | 0.0 | 0.0 |
| [$CH_2$+CO] | -43.2 | -16.6 | -13.7 | -13.0 | -11.9 | -11.2 |
| TS[$CH_2$CO] | -8.0 | 20.5 | 23.0 | 23.0 | 23.1 | 23.1 |
| [$CH_2$CO] | -41.6 | -26.1 | -20.9 | -20.1 | -18.6 | -17.3 |
| [$CH_2$CO+$H_2$] | -59.3 | -46.5 | -39.5 | -37.9 | -35.2 | -32.8 |
| TS[$CH_2$CHO] | -38.0 | -37.8 | -33.0 | -32.0 | -29.9 | -28.1 |
| [$CH_2$CHO+H] | -71.5 | -67.0 | -58.3 | -57.2 | -55.1 | -53.2 |
| TS[$CH_3$CHO] | -53.0 | -26.3 | -18.1 | -16.7 | -14.2 | -11.8 |
| [$CH_3$CHO] | -63.0 | -52.4 | -41.2 | -39.8 | -37.0 | -34.3 |
| [$CH_3$CHO+$H_2$] | -76.9 | -78.3 | -66.1 | -64.1 | -60.5 | -57.0 |
| TS[$CH_3CH_2O$] | -52.0 | -24.8 | -13.7 | -12.0 | -8.7 | -5.5 |
| [$CH_3CH_2O$+H] | -81.0 | -73.2 | -57.8 | -56.3 | -53.5 | -50.7 |
| TS[$CH_3CH_2$+O] | -42.2 | -50.6 | -39.2 | -38.5 | -37.1 | -36.0 |
| [$CH_3CH_2$+O] | -124.7 | -162.5 | -150.1 | -149.0 | -146.9 | -145.2 |

**Table S25.** Collected the absolute energies in Hartree for the for the $CH_3CH_2O$ dissociation pathway, including electronic energies at the PBEsol-D3(BJ) and B3LYP-D3(BJ)//PBEsol-D3(BJ) levels of theory, and the internal energies and Gibbs energies at the 200, 400 and 600 K at the B3LYP-D3(BJ)//PBEsol-D3(BJ) level of theory.

| System | PBEsol-D3(BJ) | B3LYP-D3(BJ)//PBEsol-D3(BJ) | | | | |
|---|---|---|---|---|---|---|
| | E | E | U | $G_{(200)}$ | $G_{(400)}$ | $G_{(600)}$ |
| [$CH_2$] | -3818.21 | -3813.14 | -3813.10 | -3813.10 | -3813.13 | -3813.17 |
| [$CH_2$+CO] | -3839.97 | -3834.80 | -3834.75 | -3834.76 | -3834.78 | -3834.82 |
| TS[$CH_2$CO] | -3839.92 | -3834.74 | -3834.69 | -3834.70 | -3834.73 | -3834.77 |
| [$CH_2$CO] | -3839.97 | -3834.82 | -3834.76 | -3834.77 | -3834.79 | -3834.83 |
| [$CH_2$CO+$H_2$] | -3841.17 | -3836.02 | -3835.95 | -3835.96 | -3835.98 | -3836.03 |
| TS[$CH_2$CHO] | -3841.14 | -3836.00 | -3835.94 | -3835.95 | -3835.98 | -3836.02 |
| [$CH_2$CHO+H] | -3841.19 | -3836.05 | -3835.98 | -3835.99 | -3836.02 | -3836.06 |
| TS[$CH_3$CHO] | -3841.16 | -3835.99 | -3835.92 | -3835.92 | -3835.95 | -3835.99 |

| | | | | | | |
|---|---|---|---|---|---|---|
| [$CH_3CHO$] | -3841.18 | -3836.03 | -3835.95 | -3835.96 | -3835.99 | -3836.03 |
| [$CH_3CHO+H_2$] | -3842.37 | -3837.24 | -3837.15 | -3837.16 | -3837.19 | -3837.23 |
| TS[$CH_3CH_2O$] | -3842.33 | -3837.15 | -3837.07 | -3837.08 | -3837.10 | -3837.15 |
| [$CH_3CH_2O+H$] | -3842.38 | -3837.23 | -3837.14 | -3837.15 | -3837.18 | -3837.22 |
| TS[$CH_3CH_2+O$] | -3842.32 | -3837.19 | -3837.11 | -3837.12 | -3837.15 | -3837.20 |
| [$CH_3CH_2+O$] | -3842.45 | -3837.37 | -3837.29 | -3837.29 | -3837.33 | -3837.37 |

## 2.5. Methanation

**Table S26.** Collected the relative energies in kcal mol$^{-1}$ for the methanation reaction pathway, including the relative electronic energies at the PBEsol-D3(BJ) and B3LYP-D3(BJ)//PBEsol-D3(BJ) levels of theory, and the relative internal energies and Gibbs energies at the 200, 400 and 600 K at the B3LYP-D3(BJ)//PBEsol-D3(BJ) level of theory.

| System | PBEsol-D3(BJ) | B3LYP-D3(BJ)//PBEsol-D3(BJ) | | | | |
|---|---|---|---|---|---|---|
| | ΔE | ΔE | ΔU | $\Delta G_{(200)}$ | $\Delta G_{(400)}$ | $\Delta G_{(600)}$ |
| [$CH_2$] | 0.00 | 0.0 | 0.0 | 0.0 | 0.0 | 0.0 |
| [$CH_2+H_2$] | -23.4 | -14.1 | -9.0 | -7.1 | -3.7 | -0.2 |
| TS[$CH_3+H$] | 7.0 | 46.8 | 49.4 | 50.0 | 51.5 | 53.2 |
| [$CH_3+H$] | -31.7 | -40.0 | -33.6 | -32.2 | -29.8 | -27.3 |
| TS[$CH_4$] | -13.6 | -2.5 | 0.1 | -1.2 | -3.1 | -5.1 |
| [$CH_4$] | -50.9 | -60.9 | -49.7 | -49.4 | -48.4 | -47.0 |

**Table S27.** Collected the absolute energies in Hartree for the methanation dissociation pathway, including electronic energies at the PBEsol-D3(BJ) and B3LYP-D3(BJ)//PBEsol-D3(BJ) levels of theory, and the internal energies and Gibbs energies at the 200, 400 and 600 K at the B3LYP-D3(BJ)//PBEsol-D3(BJ) level of theory.

| System | PBEsol-D3(BJ) | B3LYP-D3(BJ)//PBEsol-D3(BJ) | | | | |
|---|---|---|---|---|---|---|
| | E | E | U | $G_{(200)}$ | $G_{(400)}$ | $G_{(600)}$ |
| [$CH_2$] | -3818.21 | -3811.86 | -3811.82 | -3811.82 | -3811.85 | -3811.89 |
| [$CH_2+H_2$] | -3819.42 | -3813.05 | -3812.99 | -3813.00 | -3813.02 | -3813.06 |
| TS[$CH_3+H$] | -3819.37 | -3812.95 | -3812.90 | -3812.90 | -3812.93 | -3812.97 |
| [$CH_3+H$] | -3819.43 | -3813.09 | -3813.03 | -3813.04 | -3813.06 | -3813.10 |
| TS[$CH_4$] | -3819.40 | -3813.03 | -3812.98 | -3812.99 | -3813.02 | -3813.06 |
| [$CH_4$] | -3819.46 | -3813.12 | -3813.06 | -3813.06 | -3813.09 | -3813.13 |

## 2.6. Ethylene formation.

**Table S28.** Collected the relative energies in kcal mol$^{-1}$ for the ethylene formation reaction pathway, including the relative electronic energies at the PBEsol-D3(BJ) and B3LYP-D3(BJ)//PBEsol-D3(BJ) levels of theory, and the relative internal energies and Gibbs energies at the 200, 400 and 600 K at the B3LYP-D3(BJ)//PBEsol-D3(BJ) level of theory.

| System | PBEsol-D3(BJ) | B3LYP-D3(BJ)//PBEsol-D3(BJ) | | | | |
|---|---|---|---|---|---|---|
| | ΔE | ΔE | ΔU | $\Delta G_{(200)}$ | $\Delta G_{(400)}$ | $\Delta G_{(600)}$ |
| [$CH_2CH+H$] | 0.0 | 0.0 | 0.0 | 0.0 | 0.0 | 0.0 |
| TS[$CH_2CH+H$] | -28.2 | 35.0 | 34.4 | 35.0 | 36.0 | 37.1 |
| [$CH_3CH_2$] | -4.8 | -10.5 | -10.2 | -6.2 | -4.5 | -2.6 |

**Table S29.** Collected the absolute energies in Hartree for the ethylene formation reaction pathway, including electronic energies at the PBEsol-D3(BJ) and B3LYP-D3(BJ)//PBEsol-D3(BJ) levels of theory, and the internal energies and Gibbs energies at the 200, 400 and 600 K at the B3LYP-D3(BJ)//PBEsol-D3(BJ) level of theory.

| System | PBEsol-D3(BJ) | B3LYP-D3(BJ)//PBEsol-D3(BJ) | | | | |
|---|---|---|---|---|---|---|
| | E | E | U | $G_{(200)}$ | $G_{(400)}$ | $G_{(600)}$ |
| [$CH_2CH$+H] | -3841.21 | -3835.87 | -3835.80 | -3835.81 | -3835.84 | -3835.89 |
| TS[$CH_2CH$+H] | -3841.17 | -3835.81 | -3835.75 | -3835.76 | -3835.79 | -3835.83 |
| [$CH_3CH_2$] | -3841.22 | -3835.89 | -3835.82 | -3835.82 | -3835.85 | -3835.89 |

## 2.7. Ethane formation

**Table S30**. Collected the relative energies in kcal mol-1 for the ethane formation reaction pathway, including the relative electronic energies at the PBEsol-D3(BJ) and B3LYP-D3(BJ)//PBEsol-D3(BJ) levels of theory, and the relative internal energies and Gibbs energies at the 200, 400 and 600 K at the B3LYP-D3(BJ)//PBEsol-D3(BJ) level of theory.

| System | PBEsol-D3(BJ) | B3LYP-D3(BJ)//PBEsol-D3(BJ) | | | | |
|---|---|---|---|---|---|---|
| | ΔE | ΔE | ΔU | $\Delta G_{(200)}$ | $\Delta G_{(400)}$ | $\Delta G_{(600)}$ |
| [$CH_3CH_2$+H] | 0.0 | 0.0 | 0.0 | 0.0 | 0.0 | 0.0 |
| TS[$CH_3CH_2$+H] | 24.3 | 34.9 | 35.0 | 35.2 | 35.2 | 35.4 |
| [$CH_3CH_3$] | -27.3 | -27.1 | -23.1 | -23.2 | -23.4 | -23.6 |

**Table S31.** Collected the absolute energies in Hartree for the ethane formation reaction pathway, including electronic energies at the PBEsol-D3(BJ) and B3LYP-D3(BJ)//PBEsol-D3(BJ) levels of theory, and the internal energies and Gibbs energies at the 200, 400 and 600 K at the B3LYP-D3(BJ)//PBEsol-D3(BJ) level of theory.

| System | PBEsol-D3(BJ) | B3LYP-D3(BJ)//PBEsol-D3(BJ) | | | | |
|---|---|---|---|---|---|---|
| | E | E | U | $G_{(200)}$ | $G_{(400)}$ | $G_{(600)}$ |
| [$CH_3CH_2$+H] | -3842.38 | -3837.05 | -3836.96 | -3836.97 | -3837.00 | -3837.05 |
| TS[$CH_3CH_2$+H] | -3842.34 | -3836.99 | -3836.90 | -3836.91 | -3836.94 | -3836.99 |
| [$CH_3CH_3$] | -3842.42 | -3837.09 | -3837.00 | -3837.01 | -3837.04 | -3837.08 |

## 2.8. Relative barriers and thermodynamics

**Table S32.** Collected the relative barriers and thermodynamics in kcal mol$^{-1}$ for each reported reaction step for all the reaction processes considered in this work, including electronic energies at the PBEsol-D3(BJ) and B3LYP-D3(BJ)//PBEsol-D3(BJ) levels of theory, and the internal energies and Gibbs energies at the 200, 400 and 600 K at the B3LYP-D3(BJ)//PBEsol-D3(BJ) level of theory.

| System | PBEsol-D3(BJ) | | B3LYP-D3(BJ)//PBEsol-D3(BJ) | | | | | | | | | |
|---|---|---|---|---|---|---|---|---|---|---|---|---|
| | $\Delta E^{‡}$ | $\Delta E_{Rx}$ | $\Delta E^{‡}$ | $\Delta U^{‡}$ | $\Delta G^{‡}_{(200)}$ | $\Delta G^{‡}_{(400)}$ | $\Delta G^{‡}_{(600)}$ | $\Delta E_{Rx}$ | $\Delta U_{Rx}$ | $\Delta G_{Rx(200)}$ | $\Delta G_{Rx(400)}$ | $\Delta G_{Rx(600)}$ |
| TS[HCO] | 31.6 | 22.7 | 10.4 | 9.3 | 8.7 | 7.5 | 6.4 | -11.2 | -9.2 | -9.7 | -10.3 | -10.9 |
| TS[$H_2CO$] | 19.3 | 4.0 | 37.5 | 36.6 | 36.3 | 36.2 | 36.1 | 6.8 | 8.8 | 9.1 | 9.6 | 10.0 |
| TS[$H_3CO$] | 18.1 | -16.3 | 34.1 | 34.7 | 35.5 | 36.6 | 37.9 | -36.5 | -31.4 | -31.1 | -30.5 | -29.6 |
| TS[$H_3COH$] | 41.6 | 9.5 | 36.7 | 33.6 | 33.2 | 32.2 | 31.0 | -15.4 | -12.9 | -12.8 | -13.0 | -13.4 |
| | | | | | | | | | | | | |
| TS[C+O] | 88.6 | -11.6 | 93.4 | 90.3 | 88.0 | 84.7 | 81.5 | -73.6 | -74.5 | -75.0 | -76.0 | -76.9 |
| TS[HC+O] | 15.4 | -27.7 | 23.6 | 20.5 | 19.9 | 18.7 | 17.5 | -56.3 | -57.5 | -57.1 | -56.6 | -56.3 |
| TS[$H_2C$+O] | 7.6 | -31.7 | 11.3 | 9.3 | 8.7 | 7.9 | 7.0 | -34.9 | -35.8 | -36.0 | -36.1 | -36.4 |
| TS[$H_3C$+O] | 38.6 | -58.3 | 22.7 | 16.9 | 15.0 | 11.5 | 7.7 | -62.6 | -57.2 | -56.8 | -55.2 | -53.1 |
| | | | | | | | | | | | | |
| TS[$CH_2CO$] | 35.2 | 1.6 | 37.1 | 36.7 | 35.9 | 35.0 | 34.3 | -9.6 | -7.2 | -7.2 | -6.8 | -6.1 |
| TS[$CH_2CHO$] | 21.3 | -12.2 | 8.7 | 6.5 | 6.0 | 5.3 | 4.7 | -20.5 | -18.8 | -19.2 | -19.9 | -20.4 |
| TS[$CH_3CHO$] | 18.5 | 8.5 | 40.7 | 40.2 | 40.4 | 40.9 | 41.5 | 14.6 | 17.0 | 17.4 | 18.1 | 18.9 |
| TS[$CH_3CH_2O$] | 24.9 | -4.1 | 53.6 | 52.3 | 52.1 | 51.7 | 51.4 | 5.1 | 8.3 | 7.8 | 7.0 | 6.3 |
| TS[$CH_3CH_2OH$] | 29.3 | 9.3 | 31.1 | 29.9 | 29.3 | 28.4 | 27.5 | 9.5 | 13.2 | 13.3 | 13.3 | 13.3 |
| | | | | | | | | | | | | |
| TS[$CH_2C$+O] | 44.9 | -26.3 | 55.7 | 53.8 | 53.7 | 53.5 | 53.4 | -35.3 | -37.4 | -38.6 | -40.5 | -42.6 |
| TS[$CH_2CH$+O] | 44.4 | -36.3 | 24.0 | 22.5 | 22.4 | 22.5 | 22.8 | -84.9 | -88.2 | -89.2 | -91.2 | -93.4 |
| TS[$CH_3CH$+O] | 25.1 | 2.4 | 27.4 | 25.3 | 24.5 | 23.3 | 22.2 | -14.3 | -14.5 | -15.3 | -16.3 | -17.4 |
| TS[$CH_3CH_2$+O] | 38.8 | -43.8 | 22.6 | 18.6 | 17.8 | 16.3 | 14.7 | -89.3 | -92.3 | -92.7 | -93.4 | -94.5 |
| | | | | | | | | | | | | |
| TS[CH3] | 30.5 | -8.3 | 60.9 | 58.4 | 57.1 | 55.2 | 53.4 | -25.9 | -24.6 | -25.1 | -26.1 | -27.1 |
| TS[CH4] | 18.1 | -19.2 | 37.5 | 33.8 | 31.0 | 26.6 | 22.2 | -20.9 | -16.1 | -17.3 | -18.6 | -19.7 |
| | | | | | | | | | | | | |
| TS[$CH_2CH$+H] | 28.2 | -4.8 | 35.0 | -10.5 | 31.4 | -10.2 | 35.0 | -6.2 | 36.0 | -4.5 | 37.1 | -2.6 |
| TS[$CH_3CH_2$+H] | 24.3 | -27.3 | 34.9 | -27.1 | 35.0 | -23.1 | 35.2 | -23.2 | 35.2 | 23.4 | 35.4 | -23.6 |

# 3. Kinetic data

## 3.1. Arrhenius plots for the CO – first insertion path.

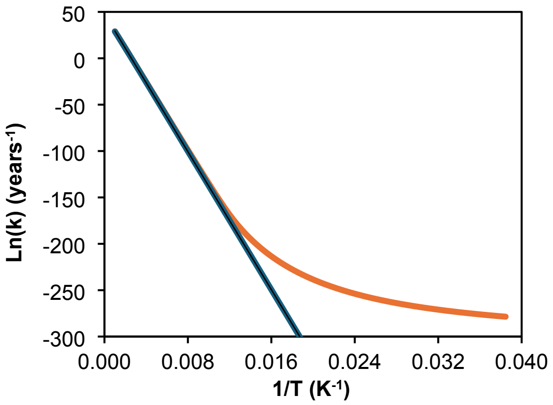


TS[HCO]

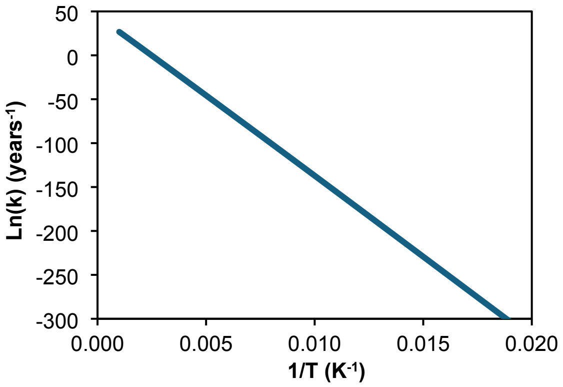


TS[$H_2CO$]

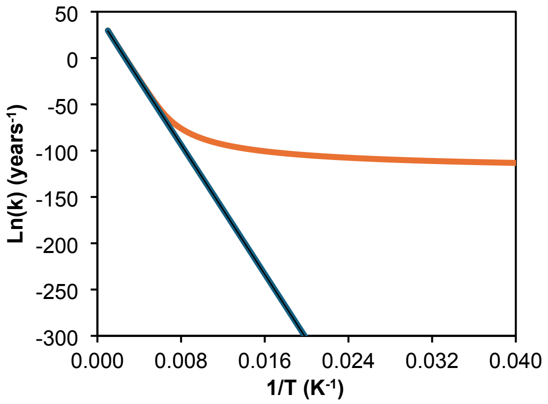


TS[$H_3CO$]

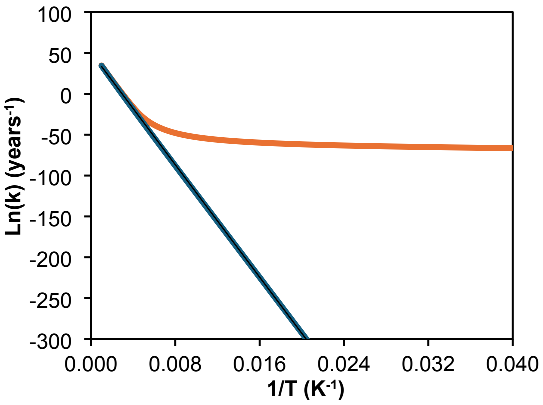


TS[$H_3COH$]

**Figure S1.** Arrhenius plots for each hydrogenation step leading to the formation of methanol in the CO – First insertion mechanism.. RRKM constants, without and considering tunnelling, are represented in blue and orange colours, respectively.

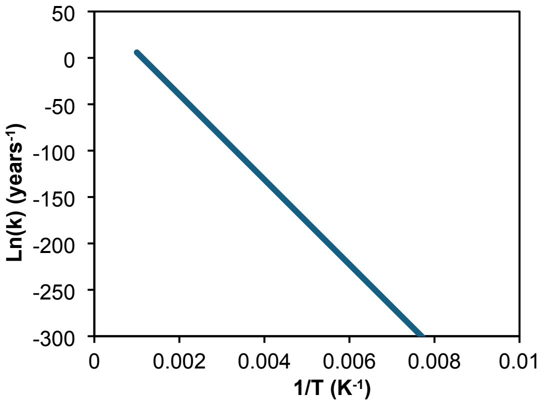


TS[C+O]

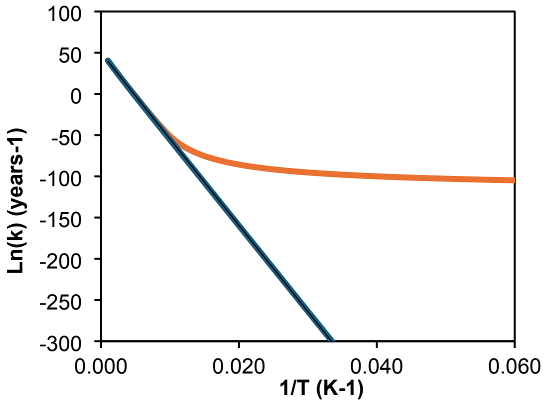


TS[HC+O]

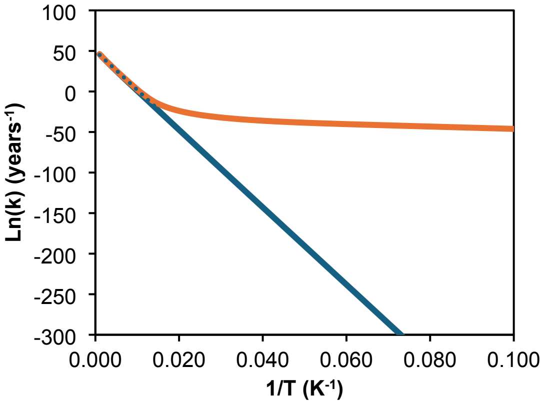


TS[$H_2C$+O]

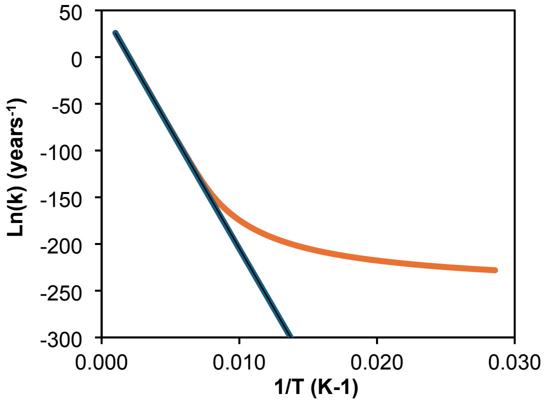


TS[$H_3C$+O]

**Figure S2.** Arrhenius plots for each CO dissociation steps in the CO – First insertion mechanism. RRKM constants, without and considering tunnelling, are represented in blue and orange colours, respectively.

### 3.2. Arrhenius plots second CO – insertion.

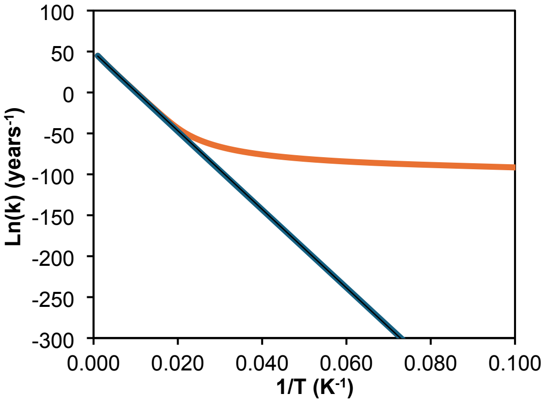


TS[$CH_2CO$]

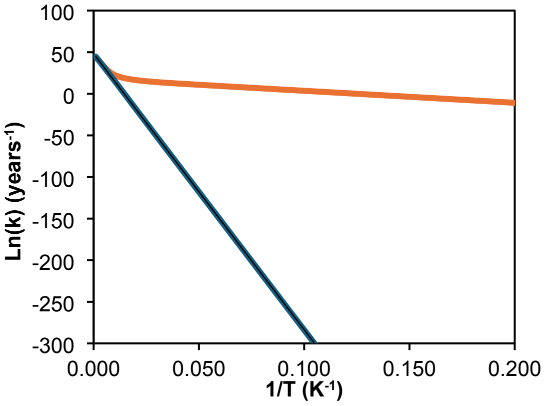


TS[$CH_2CHO$]

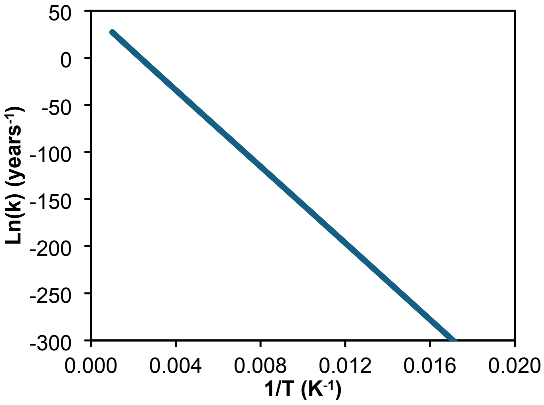


TS[$CH_3CHO$]

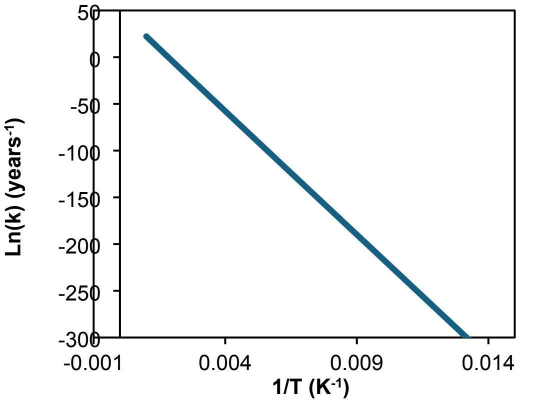


TS[$CH_3CH_2O$]

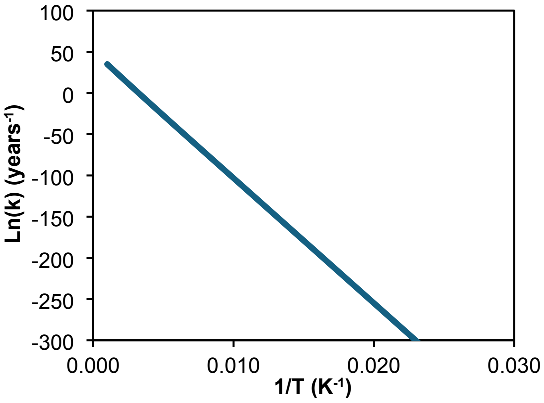


TS[$CH_3CH_2OH$]

**Figure S3.** Arrhenius plots for each hydrogenation steps leading to the formation of ethanol in the CO – Second insertion mechanism. RRKM constants, without and considering tunnelling, are represented in blue and orange colours, respectively..

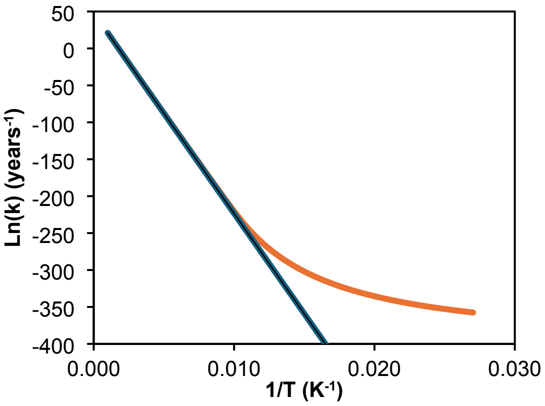


TS[$CH_2C$+O]

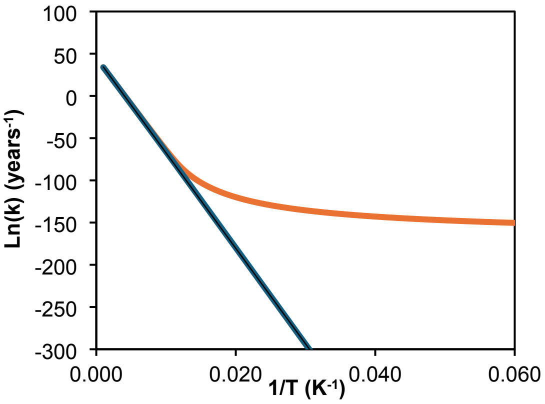


TS[$CH_2CH$+O]

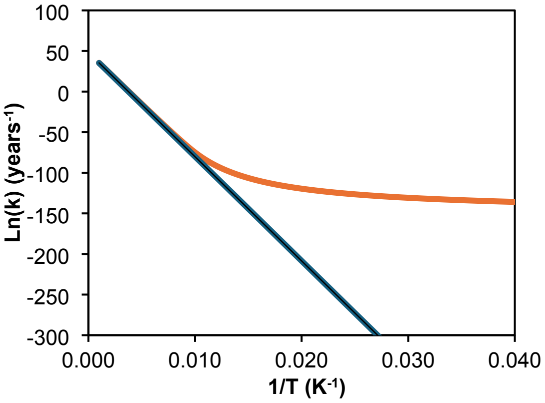


TS[$CH_3CH$+O]

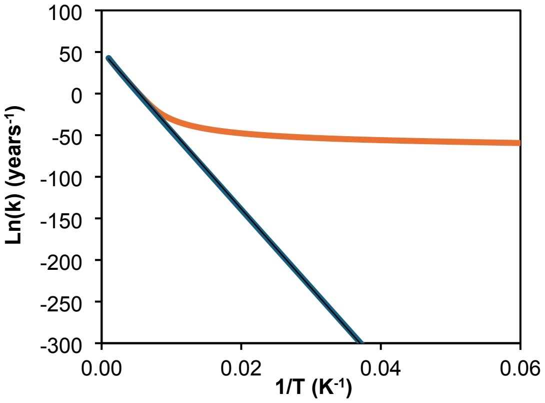


TS[$CH_3CH_2$+O]

**Figure S4.** Arrhenius plots for each CO dissociation steps in the CO – Second insertion mechanism.. RRKM constants, without and considering tunnelling, are represented in blue and orange colours, respectively.

### 3.3. Arrhenius plots methanation

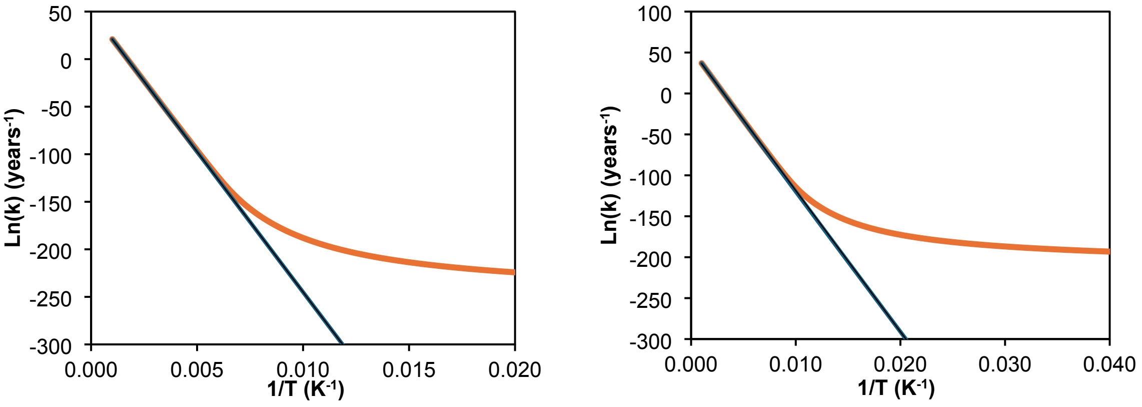


TS[$CH_3$] TS[$CH_4$]

**Figure S5.** Arrhenius plots for each hydrogenation steps leading to the formation of methane in the methanation mechanism. RRKM constants, without and considering tunnelling, are represented in blue and orange colours, respectively.

### 3.4. Arrhenius plots ethylene and ethane formation

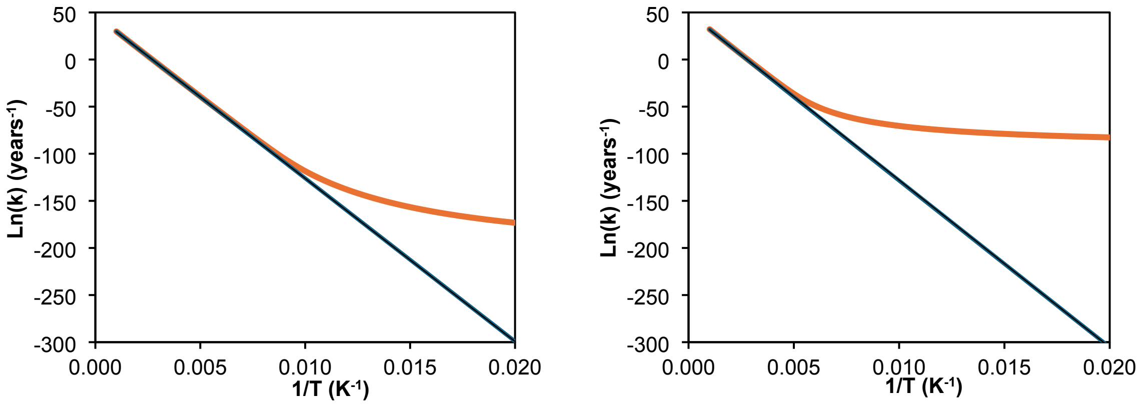


TS[$CH_2CH$ + H] TS[$CH_3CH_2$ + H]

**Figure S6.** Arrhenius plots for the hydrogenation steps leading to the formation of ethylene (leftt) and ethane (right).. RRKM constants, without and considering tunnelling, are represented in blue and orange colours, respectively.
